# Advancing at‐risk species recovery planning in an era of rapid ecological change with a transparent, flexible, and expert‐engaged approach

**DOI:** 10.1111/cobi.14421

**Published:** 2024-11-19

**Authors:** Lucas Berio Fortini, Christina R. Leopold, Fred Amidon, Devin R. Leopold, J. Scott Fretz, James D. Jacobi, Loyal Mehrhoff, Jonathan P. Price, Fern Duvall, Matthew Keir, Hank Oppenheimer, Lauren Weisenberger, Robert Sutter

**Affiliations:** ^1^ Pacific Island Ecosystems Research Center U.S. Geological Survey Hawaiʻi National Park Hawaiʻi USA; ^2^ Hawaiʻi Cooperative Studies Unit University of Hawaiʻi at Hilo Hilo Hawaiʻi USA; ^3^ Pacific Islands Fish and Wildlife Office U.S. Fish and Wildlife Service Honolulu Hawaiʻi USA; ^4^ Lafayette Colorado USA; ^5^ Division of Forestry and Wildlife Hawaiʻi Department of Land and Natural Resources Kahului Hawaiʻi USA; ^6^ Independent Researcher Tucson Arizona USA; ^7^ Geography Department University of Hawaiʻi at Hilo Hilo Hawaiʻi USA; ^8^ Division of Forestry and Wildlife Hawaiʻi Department of Land and Natural Resources Honolulu Hawaiʻi USA; ^9^ Maui Nui Plant Extinction Prevention Program Wailuku Hawaiʻi USA; ^10^ Enduring Conservation Outcomes, LLC Savannah Georgia USA

**Keywords:** climate resilience, endangered species, Hawaii, Hawaiʻi, Pacific Island, recovery planning, spatial conservation prioritization, spatial optimization, especie en peligro, Hawái, Hawai'i, Isla del Pacífico, optimización espacial, planeación de recuperación, priorización espacial de la conservación, resiliencia climática

## Abstract

In the face of unprecedented ecological changes, the conservation community needs strategies to recover species at risk of extinction. On the Island of Maui, we collaborated with species experts and managers to assist with climate‐resilient recovery planning for 36 at‐risk native plant species by identifying priority areas for the management of recovery populations. To do this, we developed a tailored spatial conservation prioritization (SCP) approach distinguished by its emphasis on transparency, flexibility, and expert (TFE) engagement. Our TFE SCP approach consisted of 2 iterative steps: first, the generation of multiple candidate conservation footprints (i.e., prioritization solutions) with a flexible greedy algorithm that reflects conservation practitioners’ priorities and, second, the selection of an optimal conservation footprint based on the consideration of trade‐offs in expert‐agreed criteria among footprints. This process maximized buy‐in by involving conservation practitioners and experts throughout, from setting goals to reviewing optimization data, defining optimization rules, and designating planning units meaningful to practitioners. We minimized the conservation footprint area necessary to meet recovery goals while incorporating species‐specific measures of habitat suitability and climate resilience and retaining species‐specific information for guiding recovery efforts. Our approach reduced the overall necessary conservation area by 36%, compared with selecting optimal recovery habitats for each species separately, and still identified high‐quality habitat for individual species. Compared with prioritizr (an existing SCP tool), our approach identified a conservation area of equal size but with higher quality habitat. By integrating the strengths of existing techniques in a flexible and transparent design, our approach can address natural resource management constraints and provide outputs suitable for local recovery planning, consequently enhancing engagement and buy‐in from conservation practitioners and experts. It demonstrates a step forward in making conservation planning more responsive to real‐world complexities and helps reduce barriers to implementation for local conservation practitioners.

## Abstract (es)

Avances en la planeación de la recuperación de especies en riesgo en una época de cambios ecológicos rápidos con un enfoque transparente y flexible con la participación de expertos

**Resumen:**

Ante los cambios ecológicos sin precedentes, la comunidad conservacionista necesita estrategias para recuperar especies en peligro de extinción. Colaboramos con expertos en especies y gestores en la isla de Maui para ayudar con la planeación de la recuperación resiliente al clima de 36 especies de plantas autóctonas en peligro mediante la identificación de áreas prioritarias para la gestión de poblaciones en recuperación. Para ello desarrollamos un enfoque de priorización espacial de la conservación (CPS) a medida que se distingue por su énfasis en la transparencia, la flexibilidad y la participación de expertos (TFE). Nuestro enfoque TFE SCP consistió en dos pasos iterativos: en primer lugar, la generación de múltiples huellas de conservación candidatas (es decir, soluciones de priorización) con un algoritmo codicioso flexible que refleja las prioridades de los profesionales de la conservación y, en segundo lugar, la selección de una huella de conservación óptima basada en la consideración de compensaciones en los criterios acordados por los expertos entre las huellas. Este proceso maximizó la implicación de los profesionales de la conservación y los expertos, desde el establecimiento de los objetivos hasta la revisión de los datos de optimización, la definición de las reglas de optimización y la designación de unidades de planificación significativas para los profesionales. Redujimos al mínimo la superficie de conservación necesaria para alcanzar los objetivos de recuperación, al tiempo que incorporamos medidas de idoneidad del hábitat y resistencia climática específicas de cada especie y conservamos información específica de cada especie para orientar los esfuerzos de recuperación. Nuestro enfoque redujo la superficie total de conservación necesaria en un 36%, en comparación con la selección de hábitats óptimos de recuperación para cada especie por separado y siguió identificando hábitats de alta calidad para especies individuales. En comparación con priorizar (una herramienta de CPS ya existente), nuestro enfoque identificó un área de conservación de igual tamaño, pero con hábitats de mayor calidad. Al integrar los puntos fuertes de las técnicas existentes en un diseño flexible y transparente, nuestro enfoque puede abordar las limitaciones de la gestión de los recursos naturales y proporcionar resultados adecuados para la planificación de la recuperación local, mejorando así el compromiso y la participación de los profesionales y expertos de la conservación. Se trata de un paso adelante para que la planificación de la conservación responda mejor a las complejidades del mundo real y contribuya a reducir las barreras que dificultan su aplicación a los profesionales locales de la conservación.

## INTRODUCTION

Conservation planning efforts are underway to recover multiple threatened and endangered species across Maui Nui (the collection of Maui, Lānaʻi, Molokaʻi, and Kahoʻolawe islands in Hawaiʻi). Although habitat conservation and recovery plans are in place for most of these species, goals remain largely unmet, and only a small fraction of species have stable or increasing populations as a result of management efforts (Fretz, 2021). Additionally, many species require not only protection of existing populations but also reintroduction to meet recovery goals. Such complexities result in a challenging multiobjective problem (Schlottfeldt et al., 2015) with ecological, climatic, economic, and logistical considerations and constraints. Reducing or ignoring these complexities is unacceptable to the conservation practitioners involved. To date, the lack of an available methodology to integrate these complexities in population‐level recovery planning for large numbers of species at fine spatial scales has hampered conservation, resulting in management driven by opportunity instead of cost‐efficient planning.

There is a wide range of spatial conservation planning tools used to identify priority conservation areas for multiple species (Marxan [Ball et al., 2009], Zonation [Moilanen, 2007], ResNet [Sarkar et al., 2009]). However, prioritization is still not widely used to guide species‐specific recovery efforts (but see Burgess et al. [2019], Sirkiä et al. [2012], and Leopold et al. [2024a]). More generally, given that spatial conservation prioritization (SCP) tools were developed to inform complex biodiversity conservation planning, there is a surprising gap between SCP research and subsequent implementation (Ball et al., 2009; Kukkala & Moilanen, 2013), although efforts are underway to more directly engage practitioners (marxanplanning.org). In a survey by Sinclair et al. (2018), 42% of SCP projects focused primarily on improving analysis techniques; less than one quarter of those also considered management implementation; and 26% of studies intended for implementation failed to include end users. Álvarez‐Romero et al. (2018) conducted a review of marine conservation planning studies and found that 35% (55) of studies were aimed at decision‐making, and one third of those failed to include end‐user participation. When included in the process, lack of transparency, lack of flexibility (Rodrigues et al., 2000), high complexity, and lack of fine‐scale outputs were cited as reasons SCP tools were unsuitable for end users (Kareksela et al., 2018; Kirlin et al., 2013; Lagabrielle et al., 2010), raising the question as to whether current tools are accessible to practitioners and suit conservation management needs.

Three main factors seem to account for this implementation gap. One potential reason for this gap is scale. SCP is often performed at regional or national scales rather than local scales needed for recovery efforts (Jones et al., 2016; Kareksela et al., 2020; Stralberg et al., 2020) (but see Leopold et al. [2024a]). This may be because SCP is largely used for reserve design, whereas population‐level recovery efforts generally occur locally. Armsworth et al. (2020) found that spatial scale choices influence SCP results, suggesting that simply downscaling broadscale analyses is not sufficient for fine‐scale efforts. The challenge becomes increasingly complex when incorporating climate‐resilient recovery efforts and associated future uncertainty (Jagannathan et al., 2021). Conservation efforts may likely benefit from plans that integrate fine‐scale spatial guidance for multiple species in a manner that facilitates complementary management across the landscape.

Another factor that may account for the implementation gap is lack of transparency in SCP tools. Transparency in conservation optimization provides insight into the reasoning and process behind the results, thereby ensuring accountability (Rodrigues et al., 2000). Practically, this translates into the ability of conservation practitioners to understand how the algorithm selects planning units (PUs) and how criteria and constraints influence the configuration of conservation footprints (i.e., prioritization solutions). SCP tools do not easily provide PU‐scale output metrics, which limits evaluation of species and site‐specific details. Although limited past work has explored how individual species influence conservation footprints (Kujala et al., 2018), these have been primarily methodological inquiries. SCP tools also typically use single‐objective approaches to solve multiobjective problems; numerous objectives and constraints are often aggregated into a single metric (Schlottfeldt et al., 2015). Although postoptimization analyses are straightforward, particularly for prioritizr (Hanson et al., 2024), users can only infer why a given PU was selected across aggregated constraints; understanding trade‐offs may be critical for directing on‐the‐ground management actions. Instead, many SCP studies have focused on finding the mathematical optima to minimize reserve area and maximize biodiversity (Kaim et al., 2017; Laitila & Moilanen, 2012; Moilanen, 2008; Pressey et al., 1996; Schuster et al., 2020). However, improved precision and more data often do not result in more efficient end results when considered by decision makers (Toomey et al., 2017). Illustrating trade‐offs across the conservation footprint and individual taxa levels may provide insights more suitable for making local management decisions and provide managers more confidence in outcomes. 

Finally, inadequate inclusion of management expertise, values, and capacity of conservation practitioners in the process is another reason for the implementation gap (Hopkinson et al., 2000; Knight et al., 2008; McNie, 2007; Toomey et al., 2017). Prioritization tools that include opportunities for conservation practitioner feedback throughout the process and flexibility to incorporate ecological and logistical realities may help bridge the gap (Jarvis et al., 2015; Kareksela et al., 2018). Although time intensive, this approach can transform regionally developed goals into defensible, fine‐scale management action plans and avoid the mismatch between researchers and conservation practitioners (Jarvis et al., 2020; Smith et al., 2009). 

With the goals and complexities of the Maui Nui recovery planning effort in mind, along with the SCP limitations identified above, we sought to develop an expert, data‐driven systematic approach to climate‐resilient, landscape‐scale conservation planning that focuses on species recovery. Unlike existing SCP tools often used for reserve design, our approach integrates population‐level planning with identification of multispecies spatially efficient conservation footprints that support the recovery of species at risk of extinction. We sought to develop an SCP approach that shifts the primary focus from mathematical and computational optima to greater transparency, flexibility, and expert (TFE) engagement and narrows the SCP implementation gap. Our TFE SCP approach is based on 2 iterative steps: first, the generation of multiple candidate conservation footprints based on a flexible greedy algorithm that allows for detailed PU selection rules that best reflect conservation practitioners’ priorities and, second, an optimal conservation footprint selection process based on a review of trade‐offs among candidate footprints based on ecological, logistical, social, and legal expert‐defined criteria followed by review and modification of the selected conservation footprint based on expert field knowledge. Our goal was to develop a transparent approach that reflects the complex decision‐making space that conservation practitioners face when choosing where to focus efforts for multispecies recovery.

## METHODS

Our TFE SCP process fits within a larger planning effort that seeks to assist conservation practitioners in determining the minimum habitat required to recover species at risk; identify priority areas for habitat management and establishment of recovery populations; identify priority areas for management efforts, such as fencing, invasive species control, and monitoring; and provide information that could inform policy and law to support species and habitat conservation. We assembled a group of experts (state land managers and botanists, federal oversight and research agencies, including the U.S. Fish and Wildlife Service [USFWS] and U.S. Geological Survey, academics, and nongovernmental organization employees) to create the plan.

We focused our work on east Maui, Hawaiʻi, and 36 associated at‐risk plant species (Leopold et al., 2023). This area covers approximately 1439 km^2^, spanning an elevation gradient from sea level to 3055 m and an annual precipitation gradient of approximately 275–8500 mm (Frazier et al., 2016).

We conducted the optimization process at the scale of polygon‐based PUs related to Hawaiian ahupuaʻa, a traditional land division scheme (Blane & Chung, 2000) that subdivides the landscape into distinct watershed‐based units (Hawaiʻi Statewide GIS Program, 2020). Aerial imagery and a digital elevation model were used to further divide PUs along natural barriers and elevational bands (Leopold et al., 2023; U.S. Geological Survey, 2017), resulting in PUs of approximately 2 km^2^. The choice of PUs (rasters, hexagons, or polygons) can influence the outcomes of SCP analyses (Nhancale & Smith, 2011). Raster grids (pixels) are commonly used due to their simplicity and compatibility with most spatial data formats, but they can lead to spatial artifacts and may not align well with natural features or administrative boundaries. Hexagons may produce more efficient and less fragmented solutions but are more complex to generate and process. Our polygon‐based approach retained geographical and habitat integrity of sites across very heterogeneous landscapes, conserved native Hawaiian place names, and allowed for easier recognition of sites by conservation practitioners.

Analysis at the PU scale offered other major advantages over conducting the analysis at the finest possible scale of spatial raster data available (250‐m pixel resolution). The 2‐km^2^ PU size was more computationally efficient and less subject to inaccuracies in fine‐scale raster data but still large enough to accommodate distinct plant populations. The configuration of PUs, where each PU has an individual name related to its watershed and elevation range, is more intuitive compared with square pixels overlaid across the landscape. This configuration was requested by conservation practitioners and thus facilitated expert engagement and input. Additionally, the topographically defined PUs are fixed and hence well suited for long‐term planning and more likely to include areas of contiguous management access. Nevertheless, just as the exact boundaries of individual raster grid cells should not be used to demarcate conservation boundaries based on standard raster‐based spatial prioritization, PU boundaries should not constrain where conservation actions take place. Actual conservation action in and around selected PUs ideally would be largely informed by logistical considerations and on‐the‐ground knowledge of conservation practitioners. Our experts preferred the term *conservation footprint* to refer to algorithm outputs over the terms *solution* and *prioritization* because it better reflected their purpose of the output and made distinction between the prioritization process and prioritization output.

### Species‐specific recovery goals and habitat data

The expert planning group generated a list of at‐risk plant taxa for recovery planning that included federally threatened and endangered species as well as select unlisted species that are rare, declining, or vulnerable to climate change. To set species‐specific recovery goals for the optimization process, we considered the number of stable populations necessary for long‐term recovery, as defined by an independent group of experts from the Hawaiʻi and Pacific Plant Recovery Coordinating Committee. We used habitat suitability models to score the quality of each species’ habitat in every PU on a scale from 0 to 1 (Amidon & Miller, 2024a). These models were reviewed by local species experts to confirm they included all known location records and adequately represented each species’ potential distribution (Amidon & Miller, 2024b). To ensure the optimization focused on the climate‐resilient portion of each species’ range, current modeled habitat suitability was clipped to only consider areas predicted to remain suitable by midcentury (2050) under 2 statistically downscaled climate scenarios based on representative concentration pathways (RCPs) 4.5 and 8.5 (Amidon & Miller, 2024b; Giambelluca et al., 2013; Timm et al., 2015).

Species’ scores were averaged at the PU scale for each species. Each resulting species’ score map was vetted by a group of botanists with local expertise. We used these maps to ensure high‐quality habitat for each species was prioritized. Additionally, modeled habitat distributions under 4 end‐of‐century climate scenarios were overlapped with each species’ current modeled habitat distribution as a metric of climate resilience to be used as a selection criteria metric in the conservation footprint selection step (described below). This climate resilience metric ranged from 0 to 4, with 0 denoting no overlap between current distribution and end‐of‐century projected distributions and 4 denoting complete overlap between current distribution and all 4 end‐of‐century projected distributions. As with species’ scores above, these values were averaged at the PU scale for each species. The 2100 predictive models included statistically and dynamically downscaled climate projections under RCPs 4.5 and 8.5 (Timm, 2017; Timm et al., 2015; Zhang et al., 2016).

### Generation of candidate conservation footprints

For our multispecies, multicriteria optimization effort, we used a greedy algorithm approach to yield 2 primary outputs: a conservation footprint for all species to be used as a guide for broadscale management efforts and a related set of priority recovery PUs for each species considered. A greedy algorithm is a problem‐solving strategy that selects the best option at each iteration until a solution is reached (Vince, 2002).

Our primary reason for using a greedy algorithm during this process was to meet all species recovery targets while minimizing the overall size of the conservation footprint. The number of stable populations needed for recovery of each species identified above was set as recovery targets in the optimization process. The process sequentially selected PUs with the largest number of species targets, iteratively updating the number of targets remaining after each PU selection. Because the algorithm potentially selects nonoptimal solutions (Simmons et al., 2019), we introduced stochasticity in the PU selection process in 2 substeps. In the first substep, PUs within a value of 5 from the maximum number of species (*n* − 5) with remaining targets were placed in a selection pool. The stochastic threshold was defined by testing multiple thresholds from 2 to 10; 5 yielded the smallest possible conservation footprints. A threshold of 10 did not result in a smaller total footprint area but rather in slightly lower mean species’ scores.

In the second substep in the selection pool described above, a PU was picked with the probability of selection being proportional to the mean species’ scores for the remaining targets. Random selection of PUs based on the weighted mean species’ score was deliberate because greedy approaches with richness‐based selection metrics disproportionately select edges of species ranges, with edge selection magnified for species with small ranges (Araújo & Williams, 2001). Because many of the species we considered in the optimization have limited distribution, it was important to include a metric that weighted for higher quality habitat. This stochastic selection process was repeated until all species targets were met. Given the iterative and transparent nature of this approach, these optimization results identified not only a general conservation footprint but also species‐specific priority recovery PUs that represent the areas in which species‐specific actions may be focused (Appendix S2).

The algorithm was repeated 200,000 times to allow for a broad exploration of potential solutions with diverse configurations. Diagnostic analyses indicated this was a conservatively larger number of runs because performing fewer than 10,000 runs yielded solutions of comparable quality to those obtained after completing 1 million runs (refer to “Candidate conservation footprint selection, review, and modification” below) (Appendix S1). A major advantage of using an optimization approach based on the greedy algorithm is that the iterative selection steps can be easily modified to best reflect the management values that guide local decision‐making. In fact, we developed and explored several additional constraints on the routine that reflected aspects of decision‐making identified by our management collaborators. These constraints resulted in a more complex, yet transparent rule set (described below) (Appendix S1). For instance, the expert planning group had a clear preference for picking areas with existing species occurrences versus areas with only modeled high habitat suitability. Hence, in the relatively simple greedy algorithm, we altered the PU selection process to reflect such a preference. This is not possible with conventional SCP tools when used in combination with the weighted suitability consideration.

Similarly, the expert planning group considered hybridization a serious risk across endangered species because outcrossing can reduce species integrity and long‐term recovery success (Fitzpatrick et al., 2015; Rhymer & Simberloff, 1996). Hence, we modified the greedy algorithm so experts could list species at risk of hybridizing in a matrix to minimize the risk of these species co‐occurring in selected PUs. Finally, experts could identify species‐specific inclusion and exclusion areas in the optimization, similar to “lock‐in” and “lock‐out” functionality in other spatial prioritization approaches (e.g., to lock out PUs with no known access from consideration). This flexibility of the underlying optimization approach counterbalanced the known deviations of the greedy algorithm process from mathematically optimal solutions, a problem that we also minimized through multiple stochastic runs (described above). Ultimately, we leveraged the simplicity of the greedy algorithm to maximize our goals of transparency and flexibility.

### Candidate conservation footprint selection, review, and modification

Given the large number of conservation footprints generated, we developed a multicriteria method to select one footprint that best matched the complex set of management and conservation values determined by the expert planning group. First, we considered 9 metrics to serve as potential selection criteria for subsequent expert review (Appendix S3). The minimum total conservation footprint area was considered to reduce the overall management area and related costs. The amount of non‐native habitat in the conservation footprint was used as a metric indicative of the amount of native habitat restoration required because most at‐risk species cannot persist in non‐native habitat. The amount of unfenced area in the footprint was another metric considered. Fences are an important, time‐consuming, and costly conservation tool widely used across Hawaiʻi that exclude extremely detrimental non‐native ungulates (Leopold & Hess, 2017). Thus, footprints with the least amount of unfenced areas may reduce recovery timelines and costs. Climate resilience was also a metric for footprint selection (described above). Land management status was considered across footprints, with the amount of land outside conservation lands reflecting the proportion of a footprint in jurisdictions or ownership status less compatible with recovery efforts. An accessibility metric included information, such as distance from roads, trails, and helipads, that represented the feasibility of on‐the‐ground recovery efforts. Spatial contiguity, a common factor across prioritization tools, was considered because habitat corridors are important for climate‐resilient conservation planning.

Correlation tests showed no substantial correlation among the metrics considered (Appendix S4). We developed histograms for each of these metrics across the 200,000 conservation footprints in order to visualize trade‐offs. After reviewing the suite of metrics, the expert planning group selected 7 metrics to be used as selection criteria and ranked them in importance. Criteria were assigned a relative weight based on their mean rank across experts (Table 1). We then scored each of the 200,000 footprints based on these combined weighted criteria and picked the footprint with the higher total score. Given the knowledge and data gaps and the uncertainties and limitations of the optimization, the selection of this single best footprint was not considered the finalized conservation footprint, but instead as a starting point for expert revisions and modifications.

**TABLE 1:** Expert‐ranked criteria used to select a priority conservation footprint after 200,000 iterations of the modified greedy algorithm.

Selection criterion	Weight^a^	Expert preference
Area outside native habitat	0.214	Minimize
Mean species’ score	0.198	Maximize
Climate resilience	0.176	Maximize
Area outside fences	0.114	Minimize
Accessibility	0.106	Maximize
Area outside conservation lands	0.102	Minimize
Contiguity	0.094	Maximize

^a^ Weighted according to the average ranked value across the expert planning group.

The expert planning group provided feedback after examining the conservation footprint, species priority maps, and metrics generated from the footprint. Experts confirmed whether the selected PUs would maximize recovery of each considered species by mid‐century and, when appropriate, suggested PUs to include or exclude from the top footprint for a species based on their local knowledge. Experts were also asked to pay special attention to single‐species PUs because their selection was driven by known records and habitat suitability values of individual species, making them more prone to inaccuracies in species records and habitat suitability models; susceptible to portions of the conservation footprint being defined by a relatively small portion of recovery targets; and more easily corrected by expert revisions. In this step, lock‐in and lock‐out of PUs could also be revised, and, if necessary, the preceding 2 steps were rerun. More broadly, to best reflect the wider recovery planning objectives described above, the optimization process incorporated expert knowledge and management goals through direct input during the optimization setup, when defining context‐specific modifications to the optimization routine, and during review and revisions of the optimization output (Appendix S5).

### Ensuring balance between spatial efficiency and optimum species habitat selection

To test whether conservation footprints properly balanced spatial efficiency with optimal species habitat selection, we compared our results with those from a process developed by USFWS to identify priority habitat for single species (Amidon & Miller, 2024b). This process considers the fine‐scale current and predicted mid‐century species habitat suitability, distribution of compatible habitat, species occurrences, and other management‐relevant factors included in our multispecies optimization. Outputs of the single‐species process were species potential recovery sites (PRS) delineated at a 250‐m resolution across the landscape that reflect the best available habitat areas for each species considered. We generalized and merged these PRS across all species to the PU scale as a single‐species optimized footprint. 

Comparison between the single‐ and multispecies optimized conservation footprints allowed us to evaluate the gains in spatial efficiency of our multispecies optimized conservation footprint; evaluate whether the multispecies optimized conservation footprint adequately represented high‐quality habitat for each species; and identify other trade‐offs in criteria used by experts in selecting a conservation footprint between our multispecies and the USFWS single‐species optimization approaches. We also used a synthetic multi species data set to compare solutions identified by our approach with mathematical optima identified by an established and widely used SCP tool, prioritizr (Hanson et al., 2024; Appendix S2). A stand‐alone R package including code used for the TFE SCP process and additional examples and vignettes are available at https://doi.org/10.5066/P137H9PF (Leopold, et al., 2024b).

## RESULTS

**FIGURE 1:**
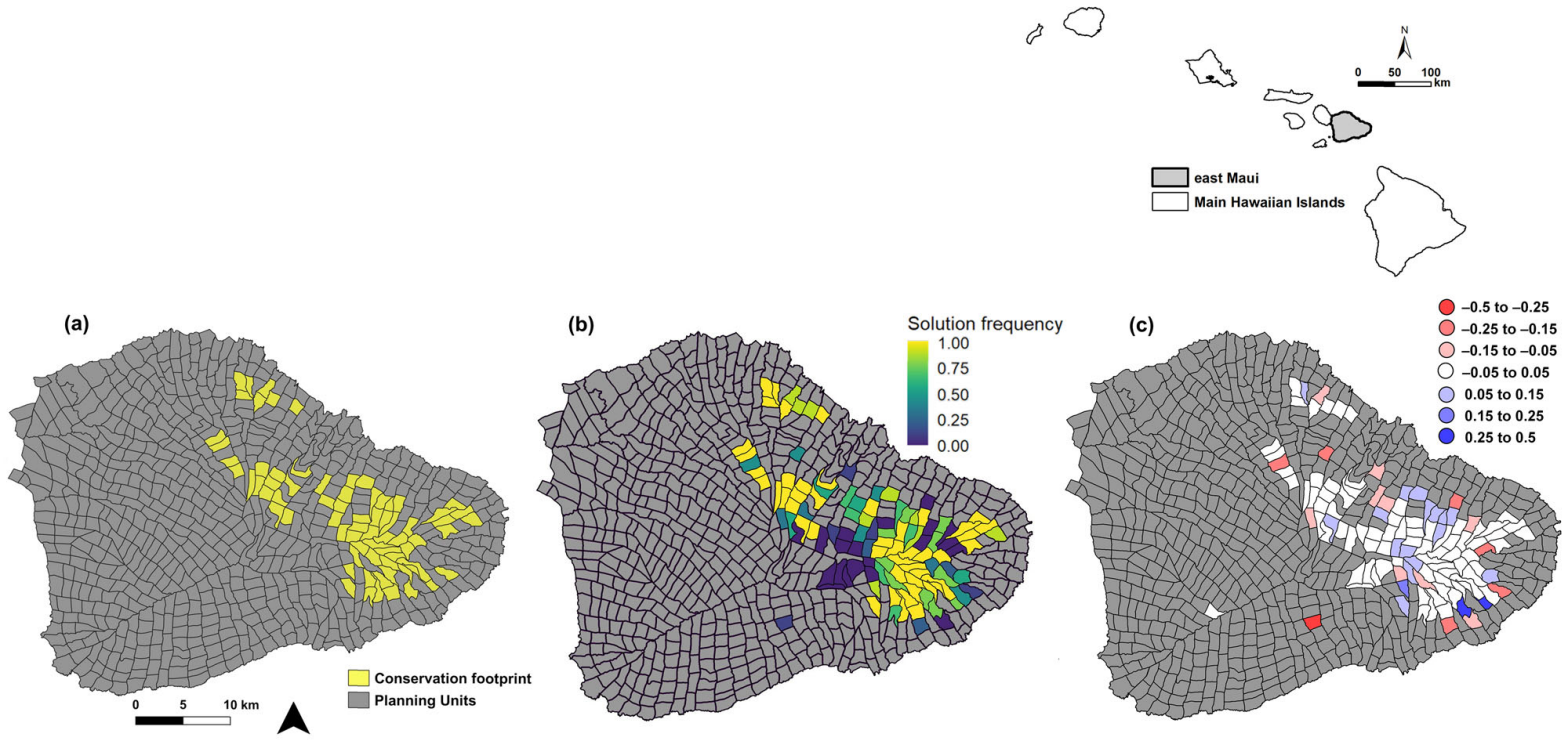
For east Maui, Hawaiʻi, the (a) conservation footprint identified using multiple selection criteria, such as species’ scores and climate resilience, (b) planning unit (PU) selection frequency across 200,000 runs, and (c) change in probability of PU inclusion given the selection frequency of the top 5% of solutions by species’ score selection criteria compared with its selection frequency across all computed solutions (blue, PU more likely to be included due to selection constraints; red, PU less likely to be included).

Of the 693 PUs we considered, 72 PUs were included in the selected conservation footprint (Figure 1a) based on the application of our TFE SCP approach. The number of target species present varied from 0 to 22 across all PUs (out of the total 36 species considered), and the maximum number of species selected for recovery in a PU was 19. The selected conservation footprint met recovery targets for all species that had sufficient initial habitat, preferentially selected PUs with existing populations, and retained climate‐resilient habitat for 31 of 36 taxa considered. The footprint included fully resilient priority recovery PUs for 60% of all species by 2100; only 7% of species priority recovery PUs had no expected climate resiliency.

### Evaluation of candidate conservation footprints

Across all 200,000 generated footprints, some PUs were consistently picked by the optimization, and others were rarely part of any solutions (Figure 1b). The PU selection frequency metric, simply calculated as the proportion of solutions that include a given PU, reflected an intuitive estimate of importance for a given PU to meeting species recovery. Our results also revealed differences in PU selection probabilities when comparing criteria‐specific top‐performing conservation footprints to the overall set of candidate conservation footprints. These differences highlighted PUs that warranted further consideration during the expert review (e.g., PUs that were much less likely to be part of any conservation footprint if the highest mean species’ scores were the primary criteria for footprint selection) (Figure 1c).

### Conservation footprint selection

The multispecies optimization approach created a footprint that was 36.8% smaller than the single‐species approach while generally still identifying high‐quality habitat for each species (Table 2). Our multispecies conservation footprint also exhibited a higher contiguity (17.9%), a 9% decrease in percent unfenced area, and a slightly more accessible (6.8%) landscape configuration compared with the single‐species optimized results. For all other metrics, comparisons showed generally small differences (<5%) between the 2 approaches. Similar species‐specific metrics showed equivalent patterns to the results across all species.

**TABLE 2:** Comparison of evaluation metrics, including all selection criteria, between the single‐species and multispecies processes used to optimize selection of recovery habitat for species at risk of extinction.

	Conservation footprint results		
Evaluation metric	TFE SCP optimization	Single‐species method	Improvement in metric (%)^a^
Area (km^2^)	140	221	36.8
Number of planning units in footprint	72	110	34.5
Mean species score	0.22	0.21	4.6
Mean habitat suitability	0.62	0.65	−4.8
Percent in good habitat	74	76	−3.5
Percent unfenced	39	43	9.3
Percent in conservation lands	92	89	3.7
Mean accessibility	2.7	2.53	6.8
Contiguity	0.04	0.03	17.9
Climate resilience	2.88	2.95	−2.5

Abbreviations: TFE SCP, transparent, flexible, and expert‐engaged spatial conservation prioritization.

^a^ Percent improvement of TFE SCP optimization over single‐species optimization. Negative values indicate single‐species method produced a preferable value.

### Expert engagement, review, and modification of the selected conservation footprint

**FIGURE 2:**
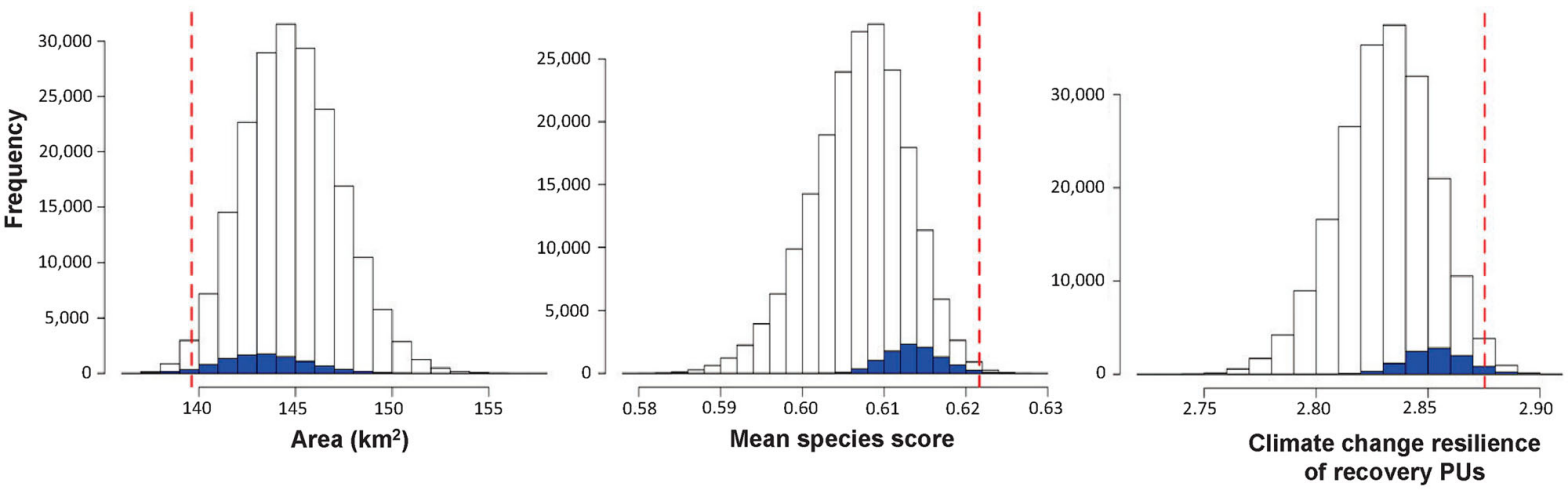
Frequency of conservation footprints relative to 3 selection criteria (white bars, all 200,000 conservation prioritization solutions; red dashed line, metric for the solution selected using the weighted criteria determined by experts; blue bars, the frequency of the top 5% of solutions based on the same criteria used to pick the best [red dashed line] solution).

**FIGURE 3:**
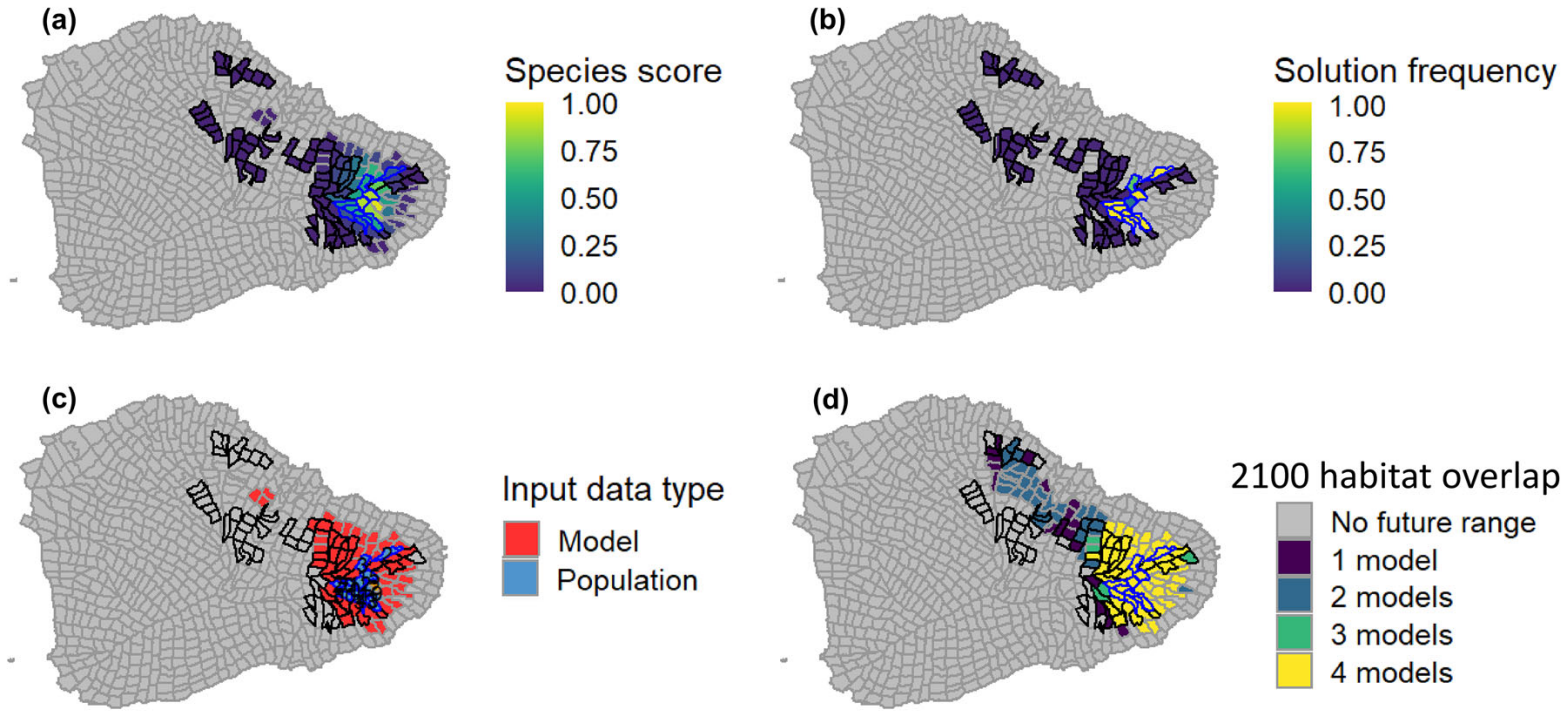
Conservation optimization outputs for *Bidens campylotheca* subsp. *waihoiensis* across east Maui, Hawaiʻi: (a) species’ score values based on habitat suitability, (b) planning unit (PU) selection frequency across 200,000 iterations, (c) type of data from which the selection occurred (modeled species’ score or species occurrence), and (d) projected habitat for the species in 2100 across 4 predictive models (black lines, conservation areas; blue lines, species priority PUs). Spatial summaries were generated for all other species for evaluation by the expert planning group. The 2100 predictive models included representative concentration pathways 4.5 and 8.5 for regionally downscaled climate projections (Timm, 2017; Timm et al., 2015; Zhang et al., 2016).

The expert planning group selected 7 metrics to be used as conservation footprint selection criteria (Appendix S3) and identified an optimal conservation footprint that balanced trade‐offs well across top selection criteria considered (Figure 2). Species‐specific outputs for each computed conservation footprint showed whether PUs were selected due to existing occurrences, habitat suitability values, or climate resilience and illustrated differences in selected species priority PUs between our optimTFE approach and the single‐species approach (Figure 3; Appendix S6). The expert planning group's review of these species priority recovery PUs allowed the experts to provide feedback by identifying problematic PUs for species and suggesting alternatives (Appendix S7). For instance, the experts’ review showed the need for the modification of species‐specific input data for *Mucuna sloanei* var. *persericea*, *Phyllostegia haliakalae*, *Phyllostegia mannii*, and *Wikstroemia villosa* to better reflect appropriate areas for species‐specific conservation.

## DISCUSSION

We developed the TFE SCP approach to create more actionable recovery plans by directly incorporating experiences of conservation practitioners across Hawaiʻi, where most recovery plans and spatial planning resources provide only general guidance for species management that ignores multiple relevant factors, such as site accessibility and existing conservation infrastructure. As such, our TFE SCP is a simple, yet comprehensive spatial optimization approach to address the complex task of identifying priority recovery areas for at‐risk taxa that minimizes the conservation footprint, incorporates climate resilience, and retains species‐specific information for guiding fine‐scale recovery efforts. By combining strengths from existing spatial prioritization techniques in a flexible design to address specific natural resource management constraints, we provide outputs at scales that suit fine‐scale recovery planning and improve process transparency for end users.

A key strength of our TFE approach lies in its ability to maximize engagement and buy‐in from on‐the‐ground conservation practitioners. Our method prioritizes the involvement of managers in every stage of the process, from goal setting and target definition to the review and modification of optimization data and solution criteria. Moreover, our flexible and transparent process allowed experts to define the underlying rules used in the optimization process, ensuring that the generated solutions align closely with real‐world management needs and constraints. This buy‐in was further enhanced by the use of PUs that reflect the way managers subdivide the landscape, making the conservation footprints not only scientifically robust but also highly applicable. Consequently, although individual technical components, such as multicriteria analysis, may not be entirely new, the value of our work lies in their cohesive integration into a framework designed to ensure practical and effective conservation outcomes. In doing so, we helped narrow key gaps that are common barriers to implementation for local conservation practitioners.

The expert planning group found it most useful to compare conservation footprints across management‐relevant criteria to fully explore potential trade‐offs. For example, when selecting a solution based on the criteria of highest mean species’ scores alone, the conservation footprint increased by 3.5% (approximately 8 km^2^) compared with the weighted criteria ultimately used. Furthermore, the conservation footprint selected using the weighted criteria covered an area 36% smaller and 17% more contiguous than the footprint based on single‐species optimization. These types of comparisons allow conservation practitioners to demonstrate effective trade‐offs when considering land acquisitions, fence installations, and staffing. Additionally, results of this process can directly inform other decision processes and associated frameworks, such as the use of Priority Threat Management (Carwardine et al., 2019), to prioritize actions and allocation of resources to maximize likelihood of species recovery.

Ensuring that results are produced at a scale useful for conservation action is critical to reducing the disconnect between research and management efforts. This optimization process was designed to allow for easy calculation of species‐ and PU‐specific metrics to evaluate conservation footprints. Moreover, it is possible to determine which species drove selection of a given PU and evaluate PU‐associated metrics. Although similar metrics can also be derived in postspatial prioritization analyses for other SCP approaches, it may not be possible to determine why a given landscape unit was selected over another, aside from instances of single target selection, because order of PU selection is not provided as output (Ball et al., 2009). Instead, most other spatial prioritization efforts focus on broad landscape scales with habitat types and biodiversity indices as evaluation metrics, producing results at a scale that cannot be easily translated to actionable effort (Cuesta et al., 2017; Jung et al., 2021; Klein et al., 2009; Kockel et al., 2020; Zhang & Li, 2022). Downscaling these broad, landscape‐scale results may lead to inaccurate inference. 

### Transparency

Because we codeveloped this approach with experts with decades of field conservation experience, it was increasingly apparent that the complexity of field conditions could not be represented in available data sets and therefore could never yield a solution that was truly optimal, regardless of the sophistication of the optimization process used (Moilanen, 2008). Hence, our approach focused on creating transparently generated footprints to serve as the basis for management and expert review and modification. This transparency is based on 3 main features: an optimization algorithm that preserves the exact PU and species selection at each iterative step in conservation footprint creation; a 2‐stage process that helps clearly identify trade‐offs across selection criteria identified by experts, aligning our work with the multicriteria analysis framework outlined by Sarkar and Illoldi‐Rangel (2010); and the inclusion of detailed species‐specific spatial prioritization information. Our method incorporates species‐specific information in terms of known occurrences and differences in habitat suitability. This is important because individual species recovery plans generally identify habitat that extends well beyond the minimum area needed for recovery, often requiring managers to identify the smaller, minimum essential habitat. Our TFE SCP approach transparently identifies the minimum essential habitat for large numbers of at‐risk species while ensuring the total conservation footprint is adequate, suitable, and sufficient to recover all species considered.

### Flexibility

Much effort in the spatial prioritization field has been placed on identifying a mathematically optimum set of solutions (Knight et al., 2008; McIntosh et al., 2018). However, heuristic approaches provide the flexibility to meet numerous, specific rule sets that can be easily defined by conservation practitioners (Moilanen, 2008; Pressey et al., 1996). This flexibility was particularly important in our work for achieving expert buy‐in and participation and outweighed the known improvements to efficiency that can be achieved using other algorithms (e.g., integer programming techniques or simulated annealing) (Ball et al., 2009; Beyer et al., 2016). Although greedy algorithm approaches have been criticized for their selection of nonoptimal solutions during simplistic minimum set problem scenarios (Possingham et al., 2000; Simmons et al., 2019; Underhill, 1994), we included a randomization allowance to reduce this possibility as suggested in Briers (2002) and demonstrate it was capable of identifying solutions with the same conservation footprint size as the prioritizr tool, which uses integer linear programming (Appendix S2). The relatively simple iterative process of a greedy search allows for customization to reflect considerations deemed crucial by conservation practitioners for developing biologically and logistically plausible conservation footprints. Additionally, the separate footprint selection step in the optimization routine allowed us to explicitly integrate multiple ecological and logistical factors important to the broader conservation planning effort, thus ensuring a proper alignment between the final approach and the decision space conservation practitioners frequently face when making similar decisions on their own. 

### Expert engagement

Spatial prioritization is a step in conservation planning where buy‐in may be challenging without properly involving practitioners. Jarvis et al. (2015) surveyed researchers and practitioners regarding the most important objectives for ensuring conservation success, and none of the top objectives included identifying efficient reserve configurations. In fact, all top‐ranked objectives included a component of cross‐disciplinary communication, for example, building community support and training to implement research recommendations, which are beyond the capacity of spatial prioritization alone. Lagabrielle et al. (2010) described an instance on Réunion Island where efforts to identify areas for biodiversity protection with a Marxan approach were rejected by decision makers because of its complexity and because values built into the tool conflicted with their beliefs. In another case, a spatial prioritization‐based plan encountered significant challenges because policy and decision makers fundamentally differed from scientists in the objectives (Kareksela et al., 2018).

It has long been recognized that it is crucial for experts to set objectives (Sarkar & Illoldi‐Rangel, 2010; Watson et al., 2011) and review and modify SCP solutions (Carwardine et al., 2009; Pressey & Bottrill, 2008). For example, Margules and Pressey (2000) state that reserve selection algorithms “provide a basis for negotiation or refinement of the conservation plan by regional or local experts.” Our coproduction approach aligns with the principles and findings of Hyman et al. (2022); increased frequency of meetings between researchers and conservation practitioners increases use of the project findings. Consequently, we found that experts and practitioners were more engaged when provided opportunities to incorporate their expertise throughout the optimization process based on their deep understanding of site conditions and ground realities, which facilitated buy‐in and improved final products. Finally, continuous expert and conservation practitioner involvement ensured that their need for clear, site‐specific products addressing species‐ and population‐level recovery action was provided at the end of the process. Production of these clearly defined data products may help bridge the implementation gap between researchers and regional management and those implementing recovery efforts.

### Caveats and limitations

In terms of computational processes, the use of a greedy heuristic algorithm can lead to unpredictable results due to the hierarchical application of rules and ties (Rodrigues et al., 2000). However, we mitigated this limitation by implementing a 2‐stage process that generates multiple solutions based on hierarchical rules and follows a multicriteria selection process defined by experts and by providing output showing which species have targets met at each PU selection during footprint generation. More broadly, despite its simplicity and flexibility, the greedy algorithm used in the first step of our process is not a computationally efficient algorithm, and its relative performance to other algorithms is likely to degrade for analyses containing very large sets of PUs. Additionally, despite the inherent simplicity of our 2‐staged approach, it still requires some technical expertise and computational resources.

In terms of expert engagement, the process of integrating feedback from conservation practitioners is time intensive (requiring over 2 years of collaboration for our case study), which potentially limits scalability and generalizability. However, engaging practitioners in defining objectives, criteria, and reviewing solutions is fundamental for relevance and applicability, allowing continuous improvement and adaptation. Furthermore, although expert involvement ensures practical relevance, it can introduce individual biases that may influence the final conservation footprint. To mitigate this, we emphasized a structured and transparent approach to expert engagement, with clearly documented and collaboratively reviewed criteria and decision‐making processes. Yet, further improvements are clearly possible, such as adapting existing expert elicitation approaches for use in the TFE SCP process (Hanea et al., 2017; Martin et al., 2012).

Although we highlight the utility of our approach in achieving the specific case study's objectives and its potential usefulness for similar planning efforts, we do not suggest replacing existing tools. Rather, we provide an alternative that may be best suited for fine‐scale species recovery planning and similar efforts. Despite the caveats and limitations described above, this is the first iteration of our approach and associated R package (Leopold, et al., 2024b), and many improvements are possible and being explored. These improvements include better visualizations and tools for expert interaction and modification, alternative rule sets, improving computational efficiency, and additional solution metrics, such as selection‐criteria‐specific irreplaceability indices (Baisero et al., 2022).

In an era of rapid ecological change, conservation practitioners are challenged to find ways to adapt species recovery to shifting climatic conditions (Lawler et al., 2015; Reside et al., 2018). Large projected range shifts are expected for many species, although there is much uncertainty and variability among regions (Nenzén & Araújo, 2011; Radeloff et al., 2015). Flexible systematic approaches to efficiently address species‐specific management of numerous taxa are needed to ensure conservation practitioners can effectively respond to shifting ecological and logistical realities.

Using our TFE SCP approach, we found that climate‐resilient priority recovery areas for at‐risk species can be identified systematically across a highly heterogeneous landscape. Because our approach was designed for prioritizing species recovery areas with direct input and evaluation by conservation practitioners, its highly modifiable structure and the ability to visualize trade‐offs provide broad application to natural resource management challenges elsewhere. In fact, we are currently applying our TFE SCP approach to identify priority recovery areas for more than 400 at‐risk species across 4 main Hawaiian Islands.

Just as there are numerous approaches in species distribution modeling tailored to a variety of specific needs, an increasing diversity of SCP approaches should benefit the field by addressing the growing complexity of conservation planning challenges in a warming world. Other efforts to tackle these complex challenges are being sought, such as the BEACON project, which integrates ecological, social, and economic considerations in systematic conservation planning (Krawchuk et al., 2012), and tools that prioritize recovery actions to maximize biodiversity recovery potential (Hanson et al., 2019; Salgado‐Rojas et al., 2023) or incorporate genetic data in reserve design (Hanson et al., 2020).

## Supporting information



Supporting information
