# Supplementary material for: Advancing at‐risk species recovery planning in an era of rapid ecological change with a transparent, flexible, and expert‐engaged approach
Source: Conserv Biol. 2024 Nov 19;39(3):e14421. doi: 10.1111/cobi.14421 (PMC12124180; doi:10.1111/cobi.14421)
Supplement: Supplementary file 1 — Supporting information [file COBI-39-e14421-s001.docx]

**SUPPORTING INFORMATION**

APPENDIX S1

**Detailed description of the TFE SCP greedy algorithm-based optimization:**

The greedy algorithm-based optimization is based on a core set of simple steps. Additional constraints are listed below the step as indicated. PU indicates planning unit.

A draft R package (greedyOpt) can be found at: <https://doi.org/10.5281/zenodo.12192230>.

- Step 1. Considering all PUs available, select top PU candidates that have the most number of **remaining** target species. In other words, the species richness index used to inform selection only considers richness of the species that do not yet have enough PUs included in the conservation footprint.
  - To reduce likelihood of non-optimal solutions, the algorithm considers PUs with as many as 5 fewer species targets than most target rich PU remaining
- Step 2. Pick single PU from candidates
  - Probability of picking PU is proportional to mean species scores, of target species, within the PU
- Step 3. Update the species target list by subtracting the targets met at PU picked
- Step 4. Repeat Steps 1-3 until number of PUs needed for each species is found
  - **Resulting selected PUs meet targets across all species**
- Step 5. Repeat process above to find multiple plausible solutions (>100k)
- Step 6. Pick solution that best meets expert defined criteria
  - Compute selection criteria metric for each solution (e.g., accessibility, least amount of new fencing required, etc.)
  - Weight selection criteria based on expert values
  - Pick solution with best combination of selection criteria as starting point for expert discussions and modifications

Steps 1-3 are repeated for the selection of each PU, as illustrated below, such that across iterations, a reduced number of species targets are considered, until all viable targets are met. In the example below, *C. peleana singulifora* will not be considered a target species from iteration i+1 and on, since its population targets have already been met.

| Species | Species targets (Iteration i) | Targets in picked PU | Species targets (Iteration i+1) |
| --- | --- | --- | --- |
| *Adenophorus periens* | 3 | 1 | 2 |
| *Asplenium peruvianum insulare* | 5 | 0 | 5 |
| *Bidens campylotheca pentamera* | 5 | 0 | 5 |
| *Bidens campylotheca waihoieniss* | 10 | 1 | 9 |
| *Clermontia oblongifolia maunsis* | 7 | 0 | 7 |
| *Clermontia peleana singuliflora* | 1 | 1 | 0 |
| *Clermontia samuelii hanaensis* | 10 | 1 | 9 |

Utilizing the simple greedy algorithm within our optimization technique offers the advantage of adaptability in iterative selection processes to conform to local management values. We developed and examined supplementary constraints to incorporate collaborator-identified decision-making aspects which culminated in a sophisticated, transparent rule set described below.

Species occurrence priority: The expert planning group strongly favored areas with confirmed species occurrences over those with solely high habitat suitability predictions, leading to a modified planning unit (PU) selection procedure within the algorithm. Including this information is optional.

In this modified approach, at each iteration (steps 1-3 above), the optimization still picks PUs with highest habitat suitability across target species, but will do so by preferentially picking within a subset of PUs with known actual occurrences if those are available.

Expanding on the previous example, the matrix below shows how, at each round of the greedy search, if a species’ remaining population target is greater than the number of PUs with occurrences, the optimization algorithm considers any suitable PUs  (PUs for a species that only have modeled habitat suitability and no occurrences) during the PU selection process.

| Species | Species targets (Iter. i) | Targets in picked PU | Species targets (Iter. i+1) | PUs with occurrences (i+1) | Suitable PUs (i+1) |
| --- | --- | --- | --- | --- | --- |
| *Adenophorus periens* | 3 | 1 | 2 | 3 | 76 |
| *Asplenium peruvianum insulare* | 5 | 0 | 5 | 3 | 33 |
| *Bidens campylotheca pentamera* | 5 | 0 | 5 | 4 | 29 |
| *Bidens campylotheca waihoiensis* | 10 | 1 | 9 | 8 | 11 |
| *Clermontia oblongifolia maunsis* | 7 | 0 | 7 | 4 | 49 |
| *Clermontia peleana singuliflora* | 1 | 1 | 0 | 0 | 84 |
| *Clermontia samuelii hanaensis* | 10 | 1 | 9 | 6 | 22 |

For instance, at the iteration i+1, the optimization can no longer consider PUs without occurrences for *Adenophorus periens*, as the number of remaining targets (2), is smaller than the number of available PUs with occurrence for the species (3).

Hybridization risk avoidance: The expert group also considered hybridization as a threat to endangered species, affecting species integrity and recovery success. Consequently, we adjusted the algorithm to allow experts to delineate species at risk of hybridization in a matrix, precluding co-occurrence in selected PUs. To implement this, at a given iteration once a PU is picked at step 2, if it has species with identified hybridization risks, then at step 3 a subroutine ensures that the PU is used to satisfy the population target of only the species with the highest habitat suitability among the set of potential hybridized species.

Expert-defined lock-in, lock-out PUs: Experts could designate species-specific inclusion and exclusion zones in the optimization. This is akin to other spatial prioritization methods.

Multi-species PUs priority: To avoid solutions that have many PUs with single species targets, at step 1 of each iteration, no PU is considered as a candidate for selection as long as there are PUs with more than one target species available. See Kujala et al. (2017) for additional information on impacts of single-species when number of features is <50.

APPENDIX S1, Table 1. List of constraints developed for use in the optimization. Not all were used in the final process. PU indicates planning unit.

| Greedy constraints | Set to: | Definition |
| --- | --- | --- |
| Stochastic threshold | 2-10 | Count below maximum target species richness willing to consider for PU selection |
| Species score minimum | 0-1 | Clips species score maps to a threshold value |
| Prioritize occurrences | T/F | Prioritize selections for PUs with existing populations or known occurrences |
| Subset PUs | T/F | T = excludes PUs from analysis using defined criteria, e.g., PUs with less than 50% of area on conservation lands |
| Hybridization risk matrix | T/F | A matrix of all species combinations; if any within the matrix = T, once selection occurs for a species, the PU is removed from consideration for the co-occuring species at risk of hybridization |
| Expert species scores modifications | .csv file | Update species-specific presence within PUs based on expert-input (occurs after first run) |
| Bioregion subset | .shp | Subset PU selection areas by bioregion |
| PUs to exclude | .csv file. | Stakeholders or experts can remove PUs from consideration throughout iterative process |

APPENDIX S1, Figure 1. Graph illustrating how criteria values change with increasing number of optimization repetitions to reach minimum footprint area, given 1,000,000 iterations.


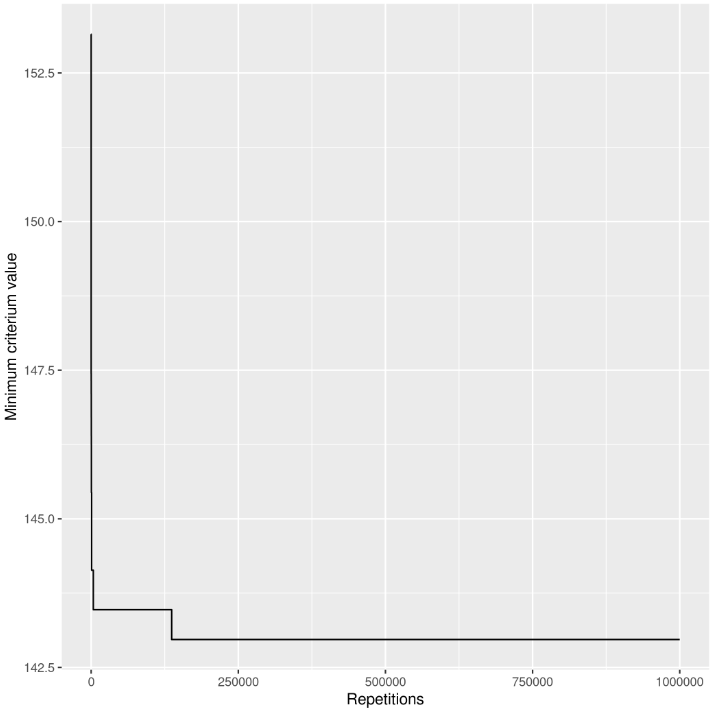


APPENDIX S1, Figure 2. Graph illustrating how criteria values change with increasing number of optimization repetitions to reach the maximum percent of conservation footprint in native habitat given 1,000,000 iterations.


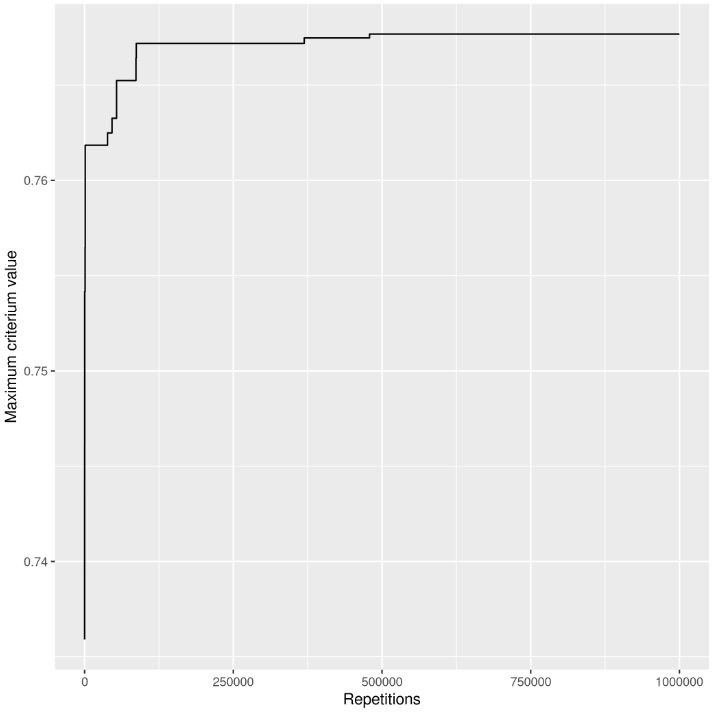


APPENDIX S1, Figure 3. Graph illustrating how criteria values change with increasing number of optimization repetitions to reach the maximum mean PU accessibility, given 1,000,000 iterations.


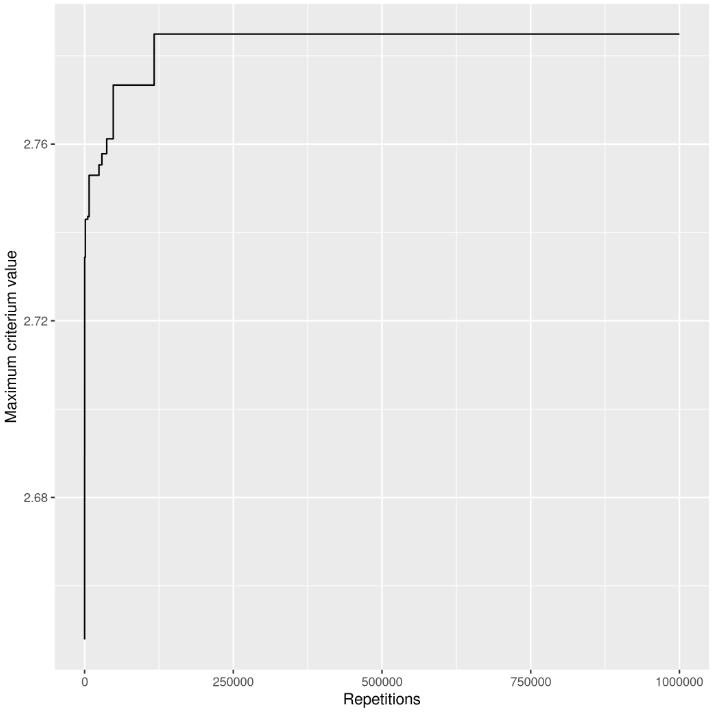


APPENDIX S1, Figure 4. Graph illustrating how criteria values change with increasing number of optimization repetitions to reach the maximum mean species scores, given 1,000,000 iterations.


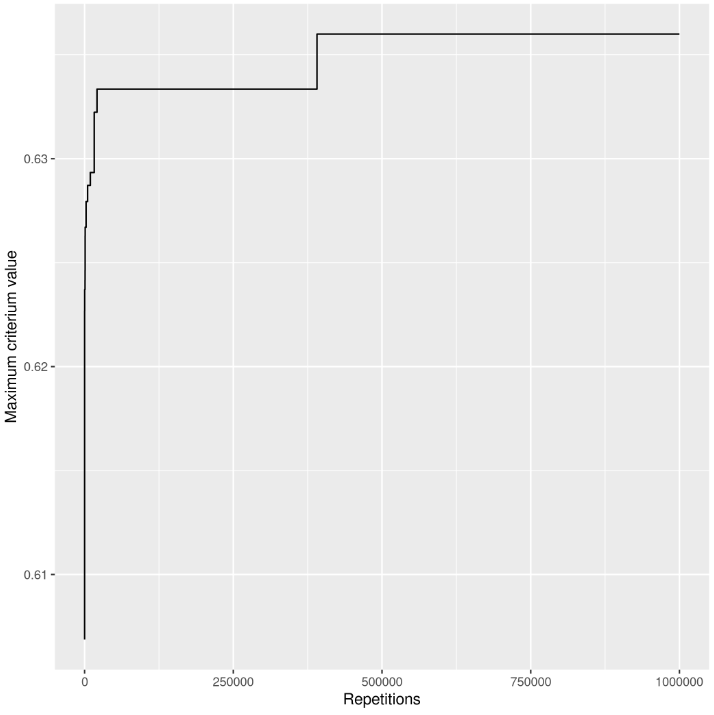


APPENDIX S1, Figure 5. Graph illustrating how criteria values change with increasing number of optimization repetitions to reach the maximum percent of conservation footprint within existing fences, given 1,000,000 iterations.


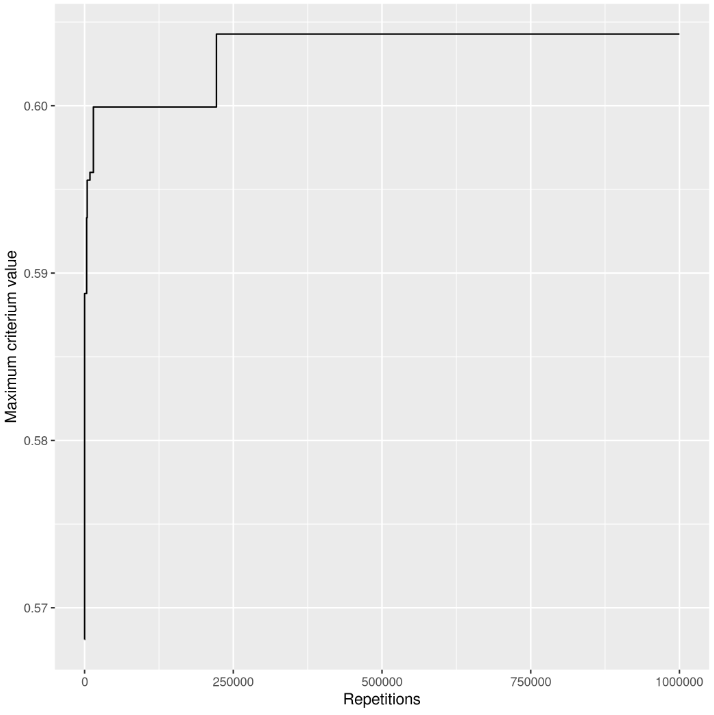


APPENDIX S1, Figure 6. Graph illustrating how criteria values change with increasing number of optimization repetitions to reach the maximum percent of conservation footprint in existing conservation lands, given 1,000,000 iterations.


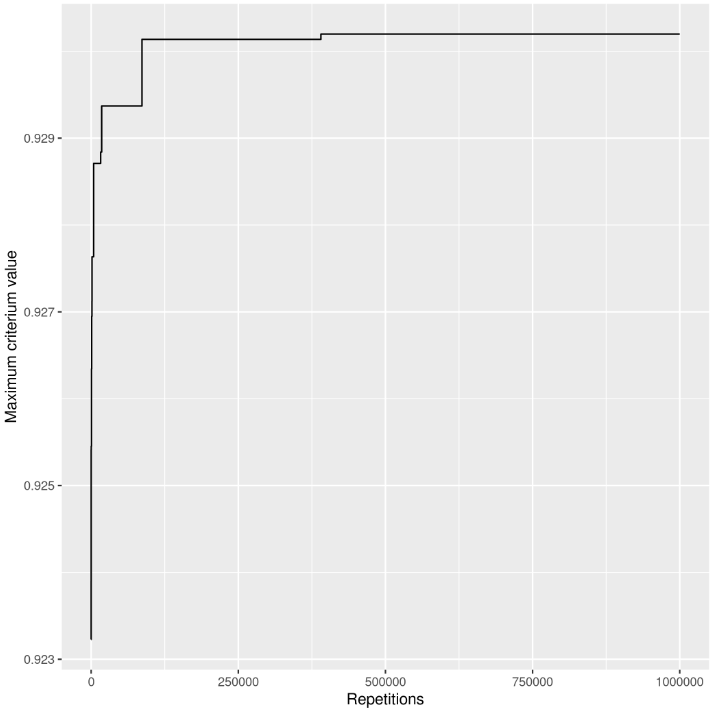


APPENDIX S1, Figure 7. Graph illustrating how criteria values change with increasing number of optimization repetitions to reach the best weighted combined 7 metric, given 1,000,000 iterations. This suite of metrics was used for conservation footprint selection. We can see that after about 70,000 repititions the criteria value is very low (good). At 500,000 repetitions we reached very low values, which did not change further upon additional repetitions.


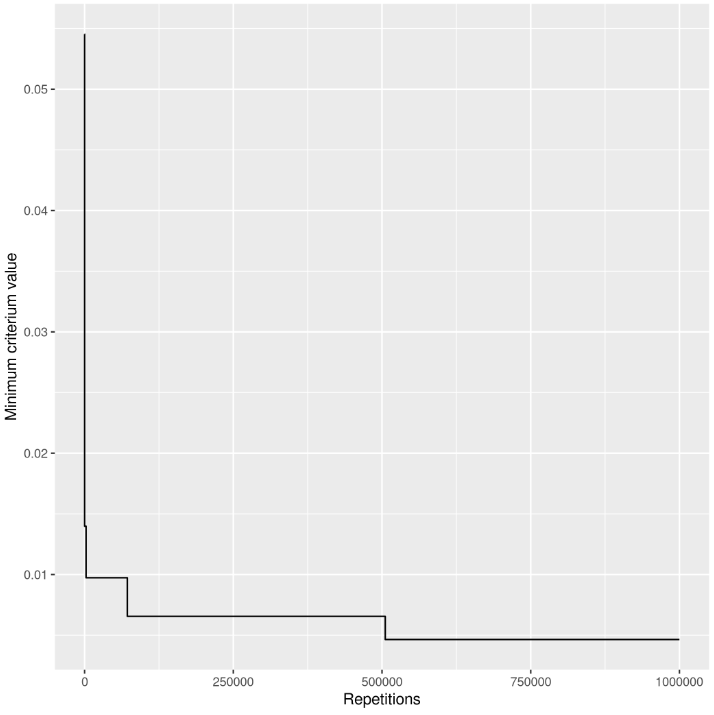


APPENDIX S2: Comparison between our approach highlighting Transparency, Flexibility and Expert Engagement (TFE SCP) and *prioritizr* (Hanson et al., 2023) using a synthetic dataset.

We created a synthetic dataset with 30 target distributions. Distributions were continuous values 0 – 1 based on mean annual temperature and rainfall of the Island of Maui (Giambelluca et al., 2013, Giambelluca et al., 2014). We binarized the target distribution data for *prioritizr* by thresholding values of ≤0.05 to 0 and remaining values to 1. While *prioritizr* can accept continuous feature values, these values cannot be used in the way we include them in our optimization process. We also thresholded values ≤0.05 to be removed from consideration in creating conservation footprints using our optimization algorithm; this eliminated values of 0 being considered technically as part of the distribution. We allowed a very large range of suitability values in order to explore potential solutions and challenge our approach to preferentially select PUs with overall higher suitability values for species in the target pool.

We set target values to 5 populations per species, and we set our TFE SCP algorithm to run 200,000 iterations with a random tolerance of 5 to match metrics used in the manuscript for the 36 east Maui plant species.

We used a gap of 0.05 to allow for a small deviation from the optimal solution, as is common in many *prioritizr* examples. We were also curious if a solution with a small gap would yield a conservation footprint that did not meet all targets but a smaller footprint. All targets were met. We ran a previous example with a gap of zero; the same conservation footprint was generated with all targets met.

Run times were approximately 1 second for *prioritizr*, while our heuristic approach took approximately 35 minutes.

Code and r package used to calculate these comparisons can be found in the online repository:  <https://doi.org/10.5281/zenodo.12192230>.

We then calculated conservation footprint summaries to evaluate the footprint size and overall habitat suitability of our solution compared to *prioritizr*. Full run parameters for each SCP approach can be found at <https://doi.org/10.5281/zenodo.12192230> in the synthetic_data folder.

Both approaches yielded a conservation footprint with the minimum size of 13 PUs, although the configurations differed.We found that there are numerous configurations of that size.

We found that habitat suitability across all species was similar between approaches, with *prioritizr* yielding a mean suitability of 0.3548 and TFE SCP 0.3594. However, when we calculated the habitat suitability score for selected features only, the TFE SCP mean suitability value rose to 0.5034.

These comparisons show that, our TFE SCP approach, despite using a simpler heuristic algorithm, can provide spatially efficient solutions comparable to those obtained from exact methods . However, if the number of species and/or planning units considered were to increase, possibly a larger number of iterations would be necessary to achieve comparable results. The TFE SCP performance above, along with clarity as to which PUs were selected for a given feature, provide conservation practitioners with feature-specific conservation footprints. It also provides enhanced flexibility to include feature-specific inputs such as habitat suitability values and prioritize across existing managed populations, and other tailored rule sets for conservation footprint creation.

Although this is a simplistic comparison, we present the shared characteristics relevant to our conservation problem between the two approaches. The TFE SCP approach can ensure known populations or locations are selected while weighting PU selection to those with higher habitat suitability within the species pool. *Prioritizr* has tremendous functionality, but it offers no straight forward way to preferentially select known populations. This approach was rejected by conservation practitioners as there was no clear way to account for habitat suitability of other species and would lead to sub-optimal recovery habitat for species with a distribution overlapping locked in areas. Furthermore, there are instances where the number of known populations is greater than the target. Habitat suitability considerations would then be needed to select the more suitable areas across species in the target pool.

These comparisons may reassure those focused on solution optimality in terms of minimum area requirements, although it is important to note that emphasis on this singular metric might be overly simplistic given the multitude of objectives beyond solution area efficiency that can be considered in SCPs, as demonstrated by our method.

APPENDIX 2, Figure 1. Output map of *prioritizr* solution that included a boundary penalty of 1, 30 features, each with a target of 5 populations. The solution required a total of 13 polygons (in yellow) to meet targets across all species.


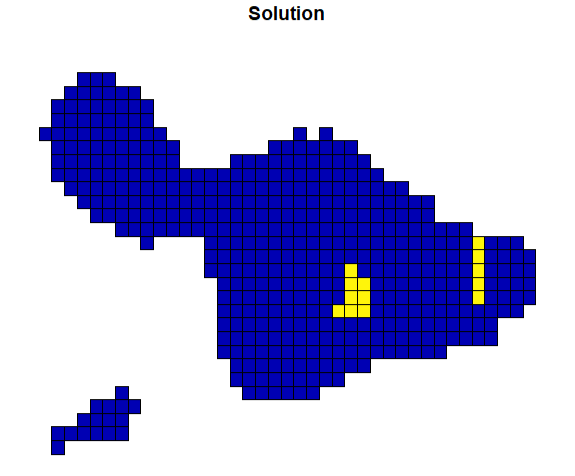


APPENDIX 2, Figure 2 Conservation footprint generated using the TFE SCP algorithm considering 30 features, each with a target of 5 populations. The randomization tolerance was set to 5, with no known occurrence or population values set, and 200,000 iterations. The solution required a total of 13 polygons (in blue) to meet targets across all species.


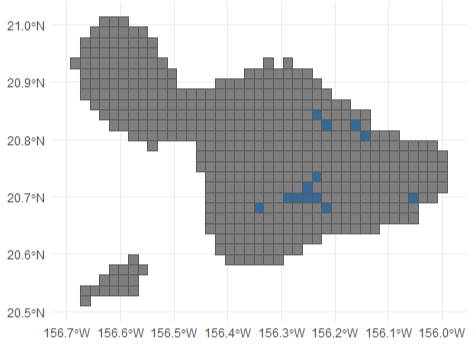


APPENDIX S2, Table 1. Mock example of output from the TFE SCP optimization algorithm. Unit_ID indicates the planning unit ID; solution is the unique solution ID across all generated; select_order indicates selection order of the planning unit (PU); sp_01, etc. represent the features considered, with a value of 1 indicating the PU was selected to include the feature and 0 indicates it was not. However, a feature may have distribution across PUs where selection value is 0. A metadata file is also generated that documents input parameters for the optimization run.

| unit_id | solution | select_order | sp_01 | sp_02 | sp_03 | sp_04 | sp_05 |
| --- | --- | --- | --- | --- | --- | --- | --- |
| 351 | 105 | 1 | 1 | 1 | 0 | 1 | 1 |
| 151 | 105 | 2 | 1 | 1 | 1 | 1 | 1 |
| 376 | 105 | 3 | 1 | 1 | 0 | 1 | 0 |
| 301 | 105 | 4 | 1 | 1 | 0 | 1 | 1 |
| 349 | 105 | 5 | 1 | 1 | 0 | 0 | 0 |
| 369 | 105 | 6 | 0 | 0 | 1 | 0 | 1 |
| 361 | 105 | 7 | 0 | 0 | 1 | 0 | 0 |
| 208 | 105 | 8 | 0 | 0 | 1 | 1 | 0 |
| 182 | 105 | 9 | 0 | 0 | 1 | 0 | 0 |
| 325 | 105 | 10 | 0 | 0 | 0 | 0 | 1 |

 APPENDIX S3. List of all selection criteria considered during the optimization process, justification for its inclusion, and data source. Contiguity and area were selection criteria, although metrics were calculated after generating a solution and is not presented here. Deer, goat and pig suitability were combined during early consideration. These input data may vary in future analyses depending on availability and project priorities. *Indicates used to select final conservation footprint.

| Map name | Purpose | Source |
| --- | --- | --- |
| PU map (planning unit) | Unique identifier for each site. This is the resolution used for selecting recovery areas to meet species targets. All conservation and species priority recovery area results are presented using PUs. | Leopold et al. 2023 |
| Species scores* | This 0-1 value applied across the study area includes habitat suitability values at the species level and are clipped to mid-century range projections. | Amidon & Miller, *in press* |
| Native/non-native habitat* | The amount of non-native habitat in conservation footprint is indicative of the amount of native habitat restoration required, as most native species cannot fully persist in non-native habitat. | Jacobi et al. 2017; https://doi.org/10.5066/F7DB80B9 |
| Fenced areas* | Indicator of protection from ungulates and the amount of area where funds and permitting required for fencing (as part of recovery) would not be required. | DLNR-DOFAW; unpublished |
| Land management status | Indicates ease of reprioritizing areas for conservation. State/federal/partner lands are more easily designated for recovery efforts than privately held lands. | Hawaiʻi Statewide GIS Program (October 2020); <https://geoportal.hawaii.gov/datasets/HiStateGIS::government-land-ownership/about> |
| Forest bird richness | Native forest bird species diversity is another interest of the planning group and areas with a higher forest bird richness would present an opportunity for management complementarity. | Fortini et al., 2015    [https://doi.org/10.1371/journal.pone.0140389](https://doi.org/10.1371/journal.pone.0140389  )  This was considered by the expert planning team, and ultimately not included as criteria in the final planning process. |
| Accessibility* | Accessibility as determined by distance from roads, trails, helipads is a logistical consideration. Preference for accessible areas would reduce transport time, establishment of roads, etc. | Hawaiʻi Statewide GIS Program (April 2020); <https://geoportal.hawaii.gov/> (April 2020) |
| Area outside of conservation lands* | Calculated to determine proportion of area already in conservation status and how much land would need repurposing to meet goals using the conservation footprint. | Hawaiʻi Statewide GIS Program (April 2020); <https://geoportal.hawaii.gov/> |
| Deer suitability | Identify areas that are likely to be negatively impacted by deer presence. | DLNR-DOFAW unpublished; see [https://dlnr.hawaii.gov/wildlife/](https://dlnr.hawaii.gov/wildlife/  )  This was considered by the expert planning team, and ultimately not included as criteria in the final planning process. |
| Goat suitability | Identify areas that are likely to be negatively impacted by goat presence. | DLNR-DOFAW unpublished; see [https://dlnr.hawaii.gov/wildlife/](https://dlnr.hawaii.gov/wildlife/  )  This was considered by the expert planning team, and ultimately not included as criteria in the final planning process. |
| Pig suitability | Identify areas that are likely to be negatively impacted by pig presence. | Risch et al. 2022  his was considered by the expert planning team, and ultimately not included as criteria in the final planning process. |

APPENDIX S4. Correlation matrix of criteria considered for selecting a conservation footprint. None of the criteria used were strongly correlated to one another. Criteria from left to right: area, area outside of native habitat, area unfenced, area outside of conservation designation, mean species score (0-1 scale), climate change resilience (0-1 scale), accessibility (ranked index), and contiguity (0-1 scale). All area criteria were in km^2^.


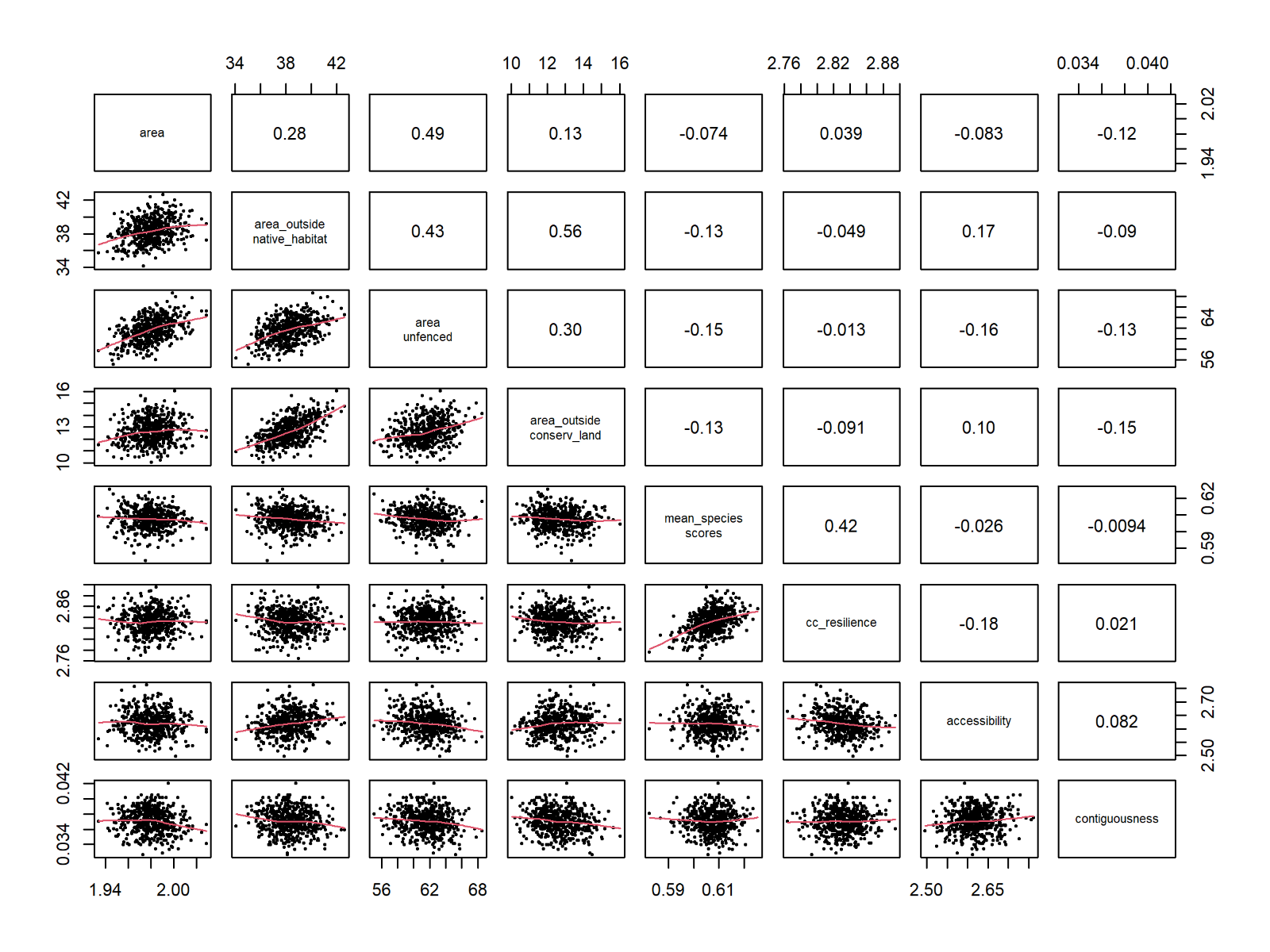


APPENDIX S5. Transparency, Flexibility and Expert Engagement (TFE) spatial prioritization process flowchart. Yellow boxes indicate process steps, blue boxes indicate intermediate products and green boxes indicate final products. The conservation footprint provides areas for focused management at the landscape scale while potential recovery areas can inform protection and recovery efforts for individual plant populations. The iterative process includes expert input and evaluation at multiple steps.


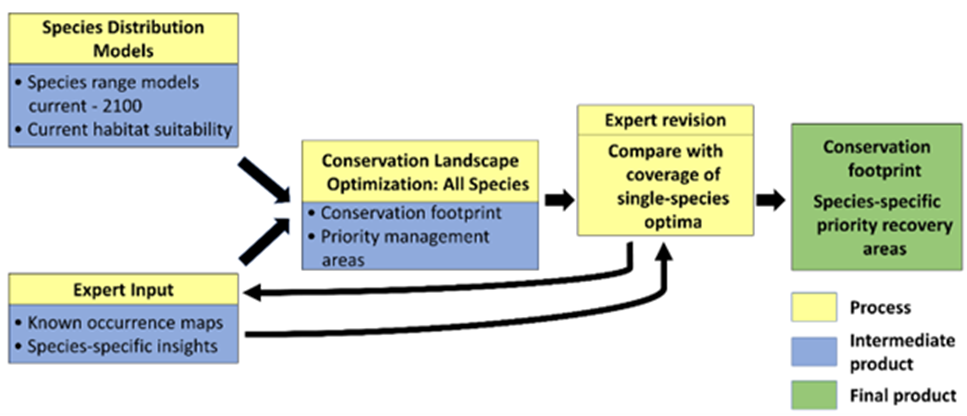


APPENDIX S6, Table 1. Table of all species included in the TFE SCP optimization, calculated metrics for two of the selection criteria for the multi-species optimization, single-species optimization (completed by US Fish & Wildlife Service), and the difference between the two. These data, along with metrics calculated across the other selection criteria, were used to identify where species-specific evaluation may be more suited for maximizing species recovery goals by 2050. Mean species score indicates habitat suitability. Climate change resilience was considered at year 2100 and four models were used for considering resilience including regionally and statistically downscaled climate projections under RCP 4.5 and 8.5 conditions (Timm, 2017; Timm et al., 2015; C. Zhang et al., 2016); values indicate the number of models in congruency. NA indicates where no species score or predictive climate models were available.

|  | Mean species score | | | Climate change resilience | | |
| --- | --- | --- | --- | --- | --- | --- |
| Species | Multi sp. optim | Single sp. optim | Diff | Multi sp. | Single sp. | Diff |
| *Adenophorus periens* | 0.293 | 0.858 | -0.565 | 4 | 4 | 0 |
| *Asplenium peruvianum* var. *insulare* | 0.9 | 0.88 | 0.02 | 2 | 2 | 0 |
| *Bidens campylotheca* subsp. *pentamera* | 0.939 | 0.756 | 0.183 | 4 | 4 | 0 |
| *Bidens campylotheca* subsp. *waihoiensis* | 0.534 | 0.567 | -0.033 | 4 | 4 | 0 |
| *Clermontia oblongifolia* subsp. *mauiensis* | 0.751 | 0.698 | 0.053 | 4 | 4 | 0 |
| *Clermontia peleana* subsp. *singuliflora* | 1 | 1 | 0 | 0 | 0 | 0 |
| *Clermontia samuelii* subsp. *hanaensis* | 0.672 | 0.748 | -0.076 | 4 | 4 | 0 |
| *Clermontia samuelii* subsp. *samuelii* | 1 | 1 | 0 | 3 | 3 | 0 |
| *Cyanea asplenifolia* | 0.717 | 0.649 | 0.068 | 4 | 4 | 0 |
| *Cyanea copelandii* subsp. *haleakalaensis* | 0.685 | 0.495 | 0.19 | 4 | 4 | 0 |
| *Cyanea duvalliorum* | 0.519 | 0.565 | -0.046 | 0.5 | 0 | 0.5 |
| *Cyanea glabra* | 0.588 | 0.588 | 0 | 4 | 4 | 0 |
| *Cyanea hamatiflora* subsp. *hamatiflora* | 0 | 0 | 0 | 1.5 | 2 | -0.5 |
| *Cyanea horrida* | 0.637 | 0.74 | -0.103 | 4 | 4 | 0 |
| *Cyanea kunthiana* | 0.739 | 0.822 | -0.083 | 4 | 4 | 0 |
| *Cyanea maritae* | 0.46 | 0.687 | -0.227 | 4 | 4 | 0 |
| *Cyanea mceldowneyi* | 0.681 | 0.72 | -0.039 | 4 | 4 | 0 |
| *Cyrtandra ferripilosa* | 0.333 | 0.333 | 0 | 2 | 2 | 0 |
| *Diplazium molokaiense* | 0.916 | 0.853 | 0.063 | 3.5 | 2.5 | 1 |
| *Geranium hanaense* | 1 | NA | NA | 0 | NA | NA |
| *Geranium multiflorum* | 0.207 | 0.343 | -0.136 | 3 | 3 | 0 |
| *Huperzia mannii* | 0.288 | 0.317 | -0.029 | 4 | 4 | 0 |
| *Joinvillea ascendens* subsp. *ascendens* | 0.812 | 0.857 | -0.045 | 4 | 4 | 0 |
| *Melicope balloui* | 0.674 | 0.723 | -0.049 | 3.5 | 3 | 0.5 |
| *Melicope ovalis* | 0.333 | 0.401 | -0.068 | 2 | 2 | 0 |
| *Menisciopsis boydiae* | 0.646 | 0.715 | -0.069 | 4 | 4 | 0 |
| *Mucuna sloanei* var. *persericea* | 1 | 1 | 0 | 3 | 2 | 1 |
| *Peperomia subpetiolata* | 1 | NA | NA | 0 | NA | NA |
| *Phyllostegia bracteata* | 0.616 | 0.598 | 0.018 | 4 | 4 | 0 |
| *Phyllostegia haliakalae* | 0.394 | 0.54 | -0.146 | 4 | 4 | 0 |
| *Phyllostegia macrophylla* | 0.964 | 0.964 | 0 | 2 | 2 | 0 |
| *Phyllostegia mannii* | 1 | 1 | 0 | 0 | 0 | 0 |
| *Phyllostegia pilosa* | 0.5 | 0.5 | 0 | 4 | 4 | 0 |
| *Platanthera holochila* | 0.858 | 0.858 | 0 | 4 | 4 | 0 |
| *Schiedea jacobii* | 0 | NA | NA | 0 | NA | NA |
| *Wikstroemia villosa* | 0.489 | 0.652 | -0.163 | 4 | 4 | 0 |

APPENDIX S6, Figure 1. Histograms of twelve selection criteria presented to the team of experts for consideration during the conservation footprint evaluation process. White bars represent all 200,000 solutions, and blue indicates the ranked top 5% of solutions. Red dashed line indicates metric for the solution selected using the weighted criteria determined by experts. The first two rows included criteria used to shape the conservation footprint while the third row was used for understanding additional factors of interest.


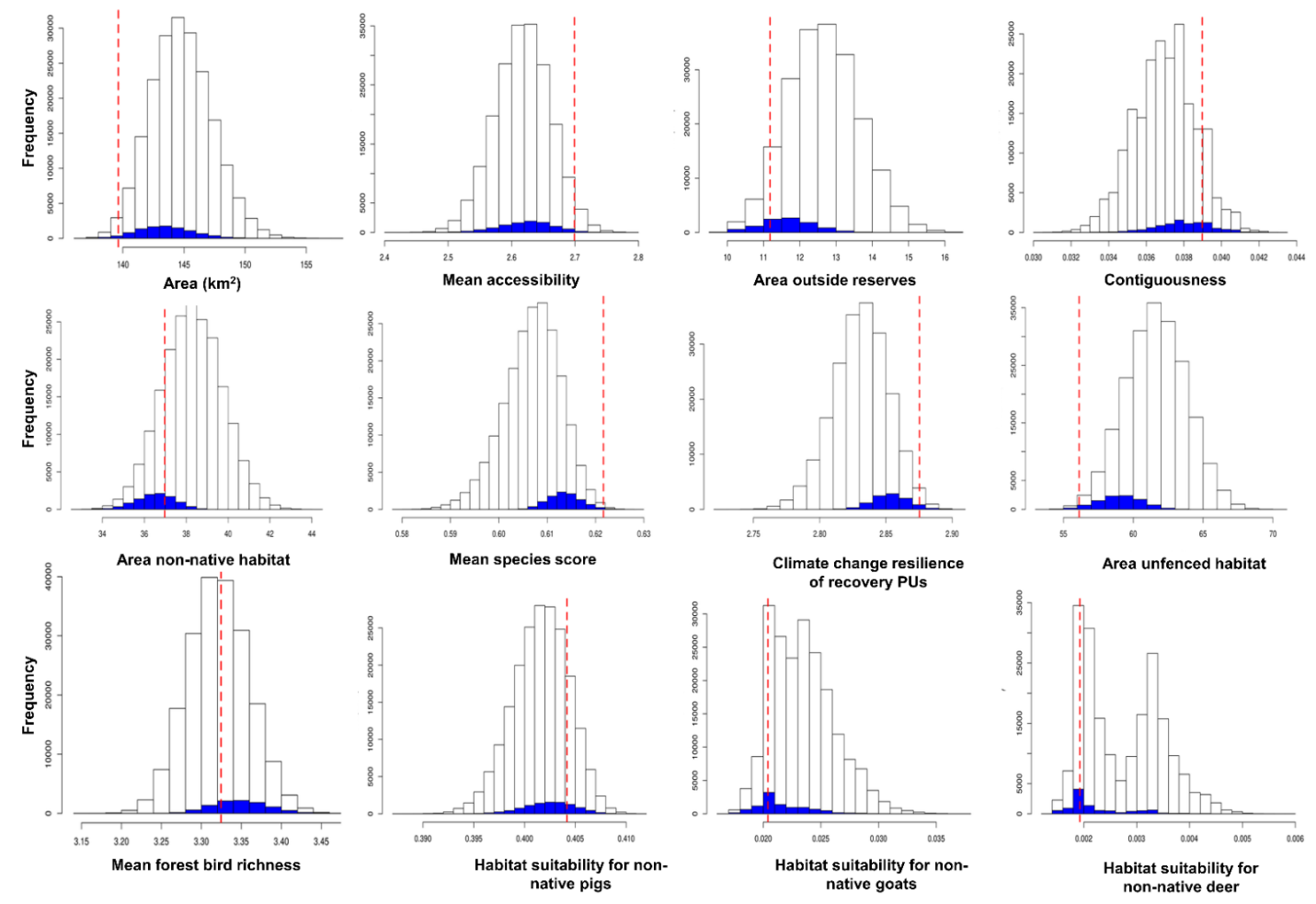


APPENDIX S6, Table 2. Metrics for each species represented in the final conservation footprint. Number PUs is the number of planning units selected for a given species based on US Fish & Wildlife Recovery guidelines. Number of existing populations for the species is listed, and the proportion of PUs selected that include existing populations. All climate resilient PUs column includes the number of PUs considered climate resilient across the 4 climate projections used to determine species ranges for 2100. The mean climate resilience across all PUs was calculated using the number of climate projections (0-4) for each PU. We also note the number of PUs with no climate resiliency for the species. For example, *Asplenium peruvianum* var. *insulare* requires 5 PUs to meet recovery goals; 5 were selected and 4 of those PUs have an existing plant or population. *A. periuvianum* has no PUs with full climate resiliency agreement across the 4 models, and the remaining PUs have anywhere from 1-3 models predicting climate resiliency in 2100.

| Species name | Number PUs | Existing pops | Proportion PUs with documented occurrence | All climate resilient PUs | Mean climate resilience | No climate resilient PUs |
| --- | --- | --- | --- | --- | --- | --- |
| *Adenophorus periens* | 3 | 0 | 0 | 3 | 4 | 0 |
| *Asplenium peruvianum* var. *insulare* | 5 | 4 | 0.80 | 0 | 2.40 | 0 |
| *Bidens campylotheca* subsp. *pentamera* | 5 | 5 | 1 | 5 | 4 | 0 |
| *Bidens campylotheca* subsp. *waihoiensis* | 10 | 10 | 1 | 10 | 4 | 0 |
| *Clermontia oblongifolia* subsp. *mauiensis* | 7 | 7 | 1 | 7 | 4 | 0 |
| *Clermontia peleana* subsp. *singuliflora* | 3 | 0 | 0 | 0 | 0 | 3 |
| *Clermontia samuelii* subsp. *hanaensis* | 10 | 7 | 0.70 | 10 | 4 | 0 |
| *Clermontia samuelii* subsp. *samuelii* | 10 | 9 | 0.90 | 0 | 2.20 | 2 |
| *Cyanea asplenifolia* | 4 | 3 | 0.75 | 4 | 4 | 0 |
| *Cyanea copelandii* subsp. *haleakalaensis* | 10 | 10 | 1 | 10 | 4 | 0 |
| *Cyanea duvalliorum* | 10 | 2 | 0.20 | 0 | 0.80 | 5 |
| *Cyanea glabra* | 3 | 2 | 0.67 | 3 | 4 | 0 |
| *Cyanea hamatiflora* subsp. *hamatiflora* | 10 | 9 | 0.90 | 0 | 1.20 | 3 |
| *Cyanea horrida* | 11 | 9 | 0.82 | 9 | 3.73 | 0 |
| *Cyanea kunthiana* | 15 | 14 | 0.93 | 14 | 3.93 | 0 |
| *Cyanea maritae* | 10 | 10 | 1 | 10 | 4 | 0 |
| *Cyanea mceldowneyi* | 10 | 9 | 0.90 | 8 | 3.80 | 0 |
| *Cyrtandra ferripilosa* | 10 | 4 | 0.40 | 3 | 2.70 | 0 |
| *Diplazium molokaiense* | 4 | 2 | 0.50 | 2 | 3.25 | 0 |
| *Geranium hanaense* | 1 | 1 | 1 | 0 | 0 | 1 |
| *Geranium multiflorum* | 11 | 9 | 0.82 | 1 | 2.73 | 0 |
| *Huperzia mannii* | 7 | 4 | 0.57 | 6 | 3.86 | 0 |
| *Joinvillea ascendens* subsp. *ascendens* | 4 | 4 | 1 | 4 | 4 | 0 |
| *Melicope balloui* | 10 | 4 | 0.40 | 5 | 3.10 | 0 |
| *Melicope ovalis* | 10 | 5 | 0.50 | 4 | 2.70 | 0 |
| *Menisciopsis boydiae* | 8 | 5 | 0.63 | 8 | 4 | 0 |
| *Mucuna persericea* | 10 | 1 | 0.10 | 4 | 3 | 0 |
| *Peperomia subpetiolata* | 1 | 1 | 1 | 0 | 0 | 1 |
| *Phyllostegia bracteata* | 10 | 5 | 0.50 | 7 | 3.40 | 0 |
| *Phyllostegia haliakalae* | 7 | 3 | 0.43 | 6 | 3.71 | 0 |
| *Phyllostegia macrophylla* | 7 | 6 | 0.86 | 0 | 1.86 | 0 |
| *Phyllostegia mannii* | 3 | 0 | 0 | 0 | 0 | 3 |
| *Phyllostegia pilosa* | 7 | 1 | 0.14 | 5 | 3.43 | 0 |
| *Platanthera holochila* | 2 | 0 | 0 | 2 | 4 | 0 |
| *Schiedea jacobii* | 1 | 1 | 1 | 0 | 0 | 1 |
| *Wikstroemia villosa* | 7 | 5 | 0.71 | 6 | 3.71 | 0 |
|  |  |  |  |  |  |  |

APPENDIX S7

While comparisons showed that our multi-species optimization was able to pick high quality habitat for most species, the ability to identify deviations by comparing to the single-species optimization was extremely valuable. For example, we observed that species priority recovery PUs picked for *Adenophorus periens* in our multi-species optimization had a lower mean species score compared to the single-species optimization, meaning suboptimal habitat was being picked for the species (APPENDIX S6). By exploring the optimization outputs, we identified that the high species richness and mean species scores of a PU in marginal *A. Periens* habitat led to its selection as a species priority recovery PU. To remedy this, our flexible approach allows us to remove those PUs from consideration for *A. periens* in the next iteration either by expert species score modification, a species-specific PU lock out, or direct solution modification by experts. This selection of species suboptimal habitat is likely to occur in other optimization approaches in similar conditions where a species with a substantially different ecological niche than the other species considered has some marginal habitat overlapping with high-quality habitat of other species. However, there is limited transparency in solutions from standard spatial prioritization tools to identify such issues because footprint-scale and single-species evaluation metrics are not outputs.

APPENDIX S7, Figure 1. Example of how species priority recovery planning units (PUs) were reviewed to identify why PUs with a lower species score were selected for *Adenophorus periens*. It is possible that ‘species-packing’ led to the selection of these PUs for *A. periens*, as the selected PUs met a high number of species recovery targets; 17, 12, and 12, respectively. Nevertheless, we can see that the PUs selected are expected to be highly resilient to end-of-century climatic conditions for the species. Blue lines indicate species priority recovery PUs while black lines indicate the conservation footprint.


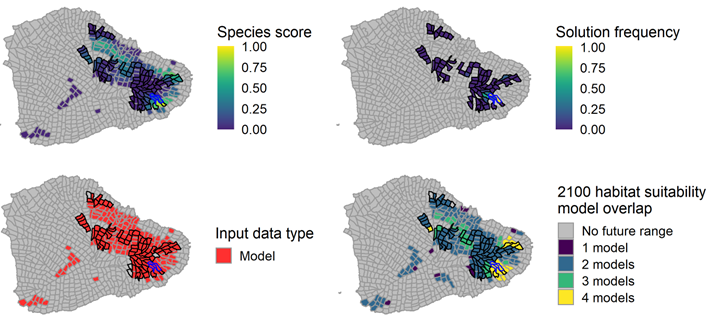


APPENDIX S8, Table 1. Comparison of several spatial conservation prioritization (SCP) tools including our TFE SCP approach, Marxan (Ball et al. 2009) and prioritizr (Hanson et al. 2023). Marxan is the most widely-used SCP tool and *prioritizr* is a more recent tool that uses exact algorithms (linear programming; Hanson et al. 2023). All tools have additional capabilities beyond those presented.

|  | **Method** | | |
| --- | --- | --- | --- |
| **Prioritization option** | **TFE SCP** | **Marxan** | ***prioritizr*** |
|  |  |  |  |
| Solution algorithm | Heuristic: Modified greedy | Simulated annealing  Iterative improvement  Heuristic options | Mixed integer linear programming |
| Feature input values | Feature inputs can range 0-1, with values serving similarly to a single-species ‘cost.’ | Feature inputs can range continuously from negative to positive values: calculations that determine if the targets are met by a solution are based on the sum of the continuous values. Over-selection occurs for the purpose of our work (Tulloch et al. 2013), but Marxan with Probability can potentially overcome such limitations (Watts et al. 2021) | Feature inputs can range continuously from negative to positive values: calculations that determine if the targets are met or not by a solution are based on the sum of the continuous values. Over-selection occurs for the purpose of our work (Tulloch et al. 2013) |
| Target setting | Can set hierarchical and/or complex selection rules; e.g. existing locations are preferentially selected over suitable habitat.  Unique targets for each feature | Unique targets can be set for each feature input. Hierarchy can be set using species penalty factor, preferentially meeting targets of some species over others | Can set hierarchical and/or complex selection rules. Unique targets can be set for each feature input. Can preferentially meet targets of some species over others by customizing weights and objective function in problem formulation |
| Cost calculations | Used to select a solution  Multiple costs permitted as selection criteria | Used to create a solution.  One cost layer permitted | Used to create a solution.  One primary cost layer permitted, but linear constraints can be used to place restrictions for additional cost layers |
| Output identifies which features determined PU selection | Yes | No | No |
| Lock-in and lock-out | Yes | Yes | Yes |
| Connectivity/contiguity consideration | Yes, contiguity can be considered as selection criteria. Solutions will first ensure all targets are met | Yes, set in problem and occurs as a penalty | Yes, set in problem and occurs as a constraint and/or penalty |
| Tradeoff evaluation | Number of criteria considered unrestricted.  See manuscript Figure 2 | Can be done generating multiple sets of solutions with different cost inputs. Challenging to consider more than two criteria at once. Uses curve plots | Can be done generating multiple sets of solutions with different cost inputs. Challenging to consider more than two criteria at once. Provides curve plots |
| Cost input: | No; second step includes selection criteria to be used in lieu of cost | Yes; cost layer input | Yes; cost layer input.  add_linear_constraints function also an option |
| Irreplaceability index | Generates metric that reflects PU selection frequency across all solutions; Can determine how shifts in selection criteria impact PU selection frequency | Generates metric that reflects PU selection frequency across all solutions; Can determine how shifts in selection criteria impact PU selection frequency | Yes, with multiple metrics available |
| Co-occurrence constraints | Yes; creates matrix where selection of one feature precludes selection of another | No | Yes, see the ‘add_linear_constraints’ tool |

**Supporting Information Literature Cited**

Amidon, F., and S. E. Miller. *In press*. Plant distribution models for the Maui Landscape Pilot Project. Technical Report. U.S. Fish and Wildlife Service, Ecological Services, Honolulu, Hawaii.

Asia-Pacific Data-Research Center [APDRC]. (2016). Statistically Downscaled Seasonal Rainfall Anomalies – Hawaiian Islands (SDSRA-HI V2). Accessed 13 April 2018 at <http://apdrc.soest.hawaii.edu/datadoc/sd5_rain.php>

Asia-Pacific Data-Research Center [APDRC]. (2017). Future projections of temperature change scenarios Downscaling of Temperature Changes for the Hawaiian Islands using CMIP5 Model Scenarios (delta-t). Accessed 13 April 2018 at <http://apdrc.soest.hawaii.edu/datadoc/sd5_delta-t.php>

Giambelluca, T.W., Q. Chen, A.G. Frazier, J.P. Price, Y.-L. Chen, P.-S. Chu, J.K. Eischeid, and D.M. Delparte. (2013). Online Rainfall Atlas of Hawai‘i. Bulletin of the American Meteorological Society 94: 313-316

Giambelluca, T.W., X. Shuai, M.L. Barnes, R.J. Alliss, R.J. Longman, T. Miura, Q. Chen, A.G. Frazier, R.G. Mudd, L. Cuo, and A.D. Businger. (2014). Evapotranspiration of Hawai‘i. Final report submitted to the U.S. Army Corps of Engineers—Honolulu District, and the Commission on Water Resource Management, State of Hawai‘i.

Hanson JO, Schuster R, Morrell N, Strimas-Mackey M, Edwards BPM, Watts ME, Arcese P, Bennett J, Possingham HP (2023). *prioritizr:* Systematic conservation prioritization in R. [https://prioritizr.net](https://prioritizr.net/), <https://github.com/prioritizr/prioritizr>.

Hijmans, R.J. (2017). raster: Geographic Data Analysis and Modeling. R package version 2.6-7. [https://CRAN.R-project.org/package=raster](https://cran.r-project.org/package=raster)

Kujala, H., Moilanen, A., Gordon, A., Travis, J. 2018. Spatial characteristics of species distributions as drivers in conservation prioritization. Methods in Ecology and Evolution 9(4) 1121-1132.

Leopold, C. R., Berio Fortini, L., Amidon, F., Fretz, S., Jacobi, J. D., Mehrhoff, L., and Sutter, R. 2023. East Maui, Hawaiʻi optimization of climate resilient habitat for native plant species recovery. (2023). U.S. Geological Survey data release, <https://doi.org/10.5066/P9LKNAR4>.

Risch, D. R., S. Honarvar, M. R. Price. 2022 Environmental drivers of seasonal shifts in abundance of wild pigs (*Sus scrofa*) in a tropical island environment. Ecological Processes 11:55 <https://doi.org/10.1186/s13717-022-00395-9>.

Timm, O.E. (2017). [Future Warming Rates over the Hawaiian Islands Based on Elevation-Dependent Scaling Factors.](https://dx.doi.org/doi:10.1002/joc.5065)International Journal of Climatology, April. doi:[10.1002/joc.5065](https://dx.doi.org/doi:10.1002/joc.5065).

Timm, O.E., T.W. Giambelluca, and H.F. Diaz. (2015). [Statistical downscaling of rainfall changes in Hawai‘i based on the CMIP5 global model projections](http://dx.doi.org/10.1002/2014JD022059). Journal of Geophysical Research Atmospheres 120: 92–112.

Tulloch, V. J., Possingham, H. P., Jupiter, S. D., Roelfsema, C., Tulloch, A. I. T., & Klein, C. J. (2013). Incorporating uncertainty associated with habitat data in marine reserve design. Biological Conservation 162 41–51.

University of Hawaii [UH]. (2013). Rainfall Atlas of Hawaii. Accessed 13 April 2018 at http://rainfall.geography.hawaii.edu/

University of Hawaii [UH]. 2014. Climate of Hawaii. Accessed 13 April 2018 at <http://climate.geography.hawaii.edu/>

Watts, M. E., I. R. Ball, S. S. Romola, C. J. Klein, K. Wilson, C. Steinback, R. Lourival, L. Kircher, and Hugh P. Possingham. (2009). Marxan with Zones: Software for optimal conservation based land- and sea-use zoning, Environmental Modelling & Software, Volume 24, Issue 12, pg. 1513-1521. <https://doi.org/10.1016/j.envsoft.2009.06.005>.

Watts, M., Klein, C. J., Tulloch, V. J., Carvalho, S. B., & Possingham, H. P. (2021). Software for prioritizing conservation actions based on probabilistic information. Conservation Biology 35(4) 1299–1308.

Zhang, L., & Li, J. (2022). Identifying priority areas for biodiversity conservation based on Marxan and InVEST model. Landscape Ecology, 37(12), 3043–3058. <https://doi.org/10.1007/s10980-022-01547-0>
